# Long-term treatment with benzodiazepines and related Z-drugs exacerbates breast cancer: clinical evidence and molecular mechanisms

**DOI:** 10.1186/s11658-025-00752-4

**Published:** 2025-06-29

**Authors:** Wei-Chung Vivian Yang, Yen-Yi Lin, Jeak Ling Ding, Chin-Sheng Hung, Phung-Anh Nguyen, Bo-Xiang Zhang, Tsung-Han Hsieh, Shu-Chun Chang

**Affiliations:** 1 The Ph.D. Program for Translational Medicine, College for Medical Science and Technology, Taipei Medical University, Taipei, 110 Taiwan; 2 International Ph.D. Program for Translational Science, College of Medical Science and Technology, Taipei Medical University, Taipei, 110 Taiwan; 3 Reproductive Medicine Center, Taipei Medical University Hospital, Taipei, 110 Taiwan; 4 Department of Biological Sciences, National University of Singapore, Singapore, 117543 Singapore; 5 Division of Breast Surgery, Department of Surgery, Taipei Medical University Hospital, Taipei, 110 Taiwan; 6 Division of General Surgery, Department of Surgery, School of Medicine, College of Medicine, Taipei Medical University, Taipei, 110 Taiwan; 7 Clinical Data Center, Office of Data Science, Taipei Medical University, Taipei, 110 Taiwan; 8 Clinical Big Data Research Center, Taipei Medical University Hospital, Taipei Medical University, Taipei, 110 Taiwan; 9 Research Center of Health Care Industry Data Science, College of Management, Taipei Medical University, Taipei, 110 Taiwan; 10 Graduate Institute of Data Science, College of Management, Taipei Medical University, Taipei, 110 Taiwan; 11 Ji Yan Biomedical (JY BioMed) Co., Ltd, Taipei, 110 Taiwan; 12 Precision Health Center, Taipei Medical University, Taipei, 110 Taiwan; 13 TMU Research Center of Cancer Translational Medicine, Taipei Medical University, Taipei, 110 Taiwan; 14 Yu Chun Biotech Co., Ltd, Taipei, 110 Taiwan

**Keywords:** Benzodiazepines and Z-drugs, Breast cancer, Extracellular matrix, GABA receptors, Clinical database informatics, Tumor microenvironment, CRISPR/Cas9 strategy

## Abstract

**Background:**

Benzodiazepines (Diazepam) and related Z-drugs (Zolpidem), henceforth referred to as BZDRs, are widely used for clinical treatment of insomnia and anxiety disorders. BZDRs act on GABA type A receptors to inhibit neurotransmitters. We previously demonstrated that prolonged clinical use of BZDRs exacerbates the risk of breast cancer (BRCA).

**Methods:**

By biomedical, health informatics platform analyses and in vivo studies, we explored clinical association between BZDR usage and BRCA development and advancement. Furthermore, by retrospective studies on patient clinical data and in vitro empirical analyses of the impact of BZDR on BRCA cells, and together with ingenuity pathway analysis (IPA) analyses, we validated the signaling pathways and identified potential intermolecular crosstalk involved.

**Results:**

Clinical data showed that BRCA patients on long term treatment with BZDRs suffered increased mortality rate (p = 0.034). Studies on patient samples indicated that among 16 GABA receptors examined, GABRA3 (a pro-tumorigenic player) was significantly upregulated by BZDRs, which advanced BRCA disease. To support our clinical findings, we examined in vivo*,* the impact of BZDRs on BRCA advancement using MDA-MB231 cells to mediate metastasis in mice model. Our results show that BZDRs indeed promoted cancer advancement to the lungs and localized in the tibia. Using BRCA cell lines, we revealed the molecular-cellular effects of prolonged treatment with BZDRs in vitro. We showed significant metastasis indicated by increased cancer cell migration and invasion, which correlated well with our clinical observations. We discovered that BZDR-mediated GABRA3 stimulation was associated with downregulation of anti-tumorigenic extracellular matrix (ECM) molecules (S100B, COL6A6 and VIT) and upregulation of pro-tumorigenic FBN3 in BRCA cells. Notably, GABRA3-shRNA and GABRA3-CRISPR/Cas9 disrupted the abovementioned dynamics dramatically and suppressed BRCA cell invasion induced by BZDRs. Bioinformatics analyses highlighted molecular pathways showing interplay between GABRA3 and ECMs, which presumably exacerbated BZDR-induced BRCA progression via immune modulators.

**Conclusions:**

Long-term clinical use of BZDRs significantly increased the mortality rate of BRCA patients. We provide in vivo and in vitro evidence confirming that BZDRs promote BRCA advancement. We revealed that BZDR-mediated BRCA signaling pathways through GABRA3-ECMs, which promotes metastasis, probably through immune modulation and changes in the tumor microenvironment.

**Graphical Abstract:**

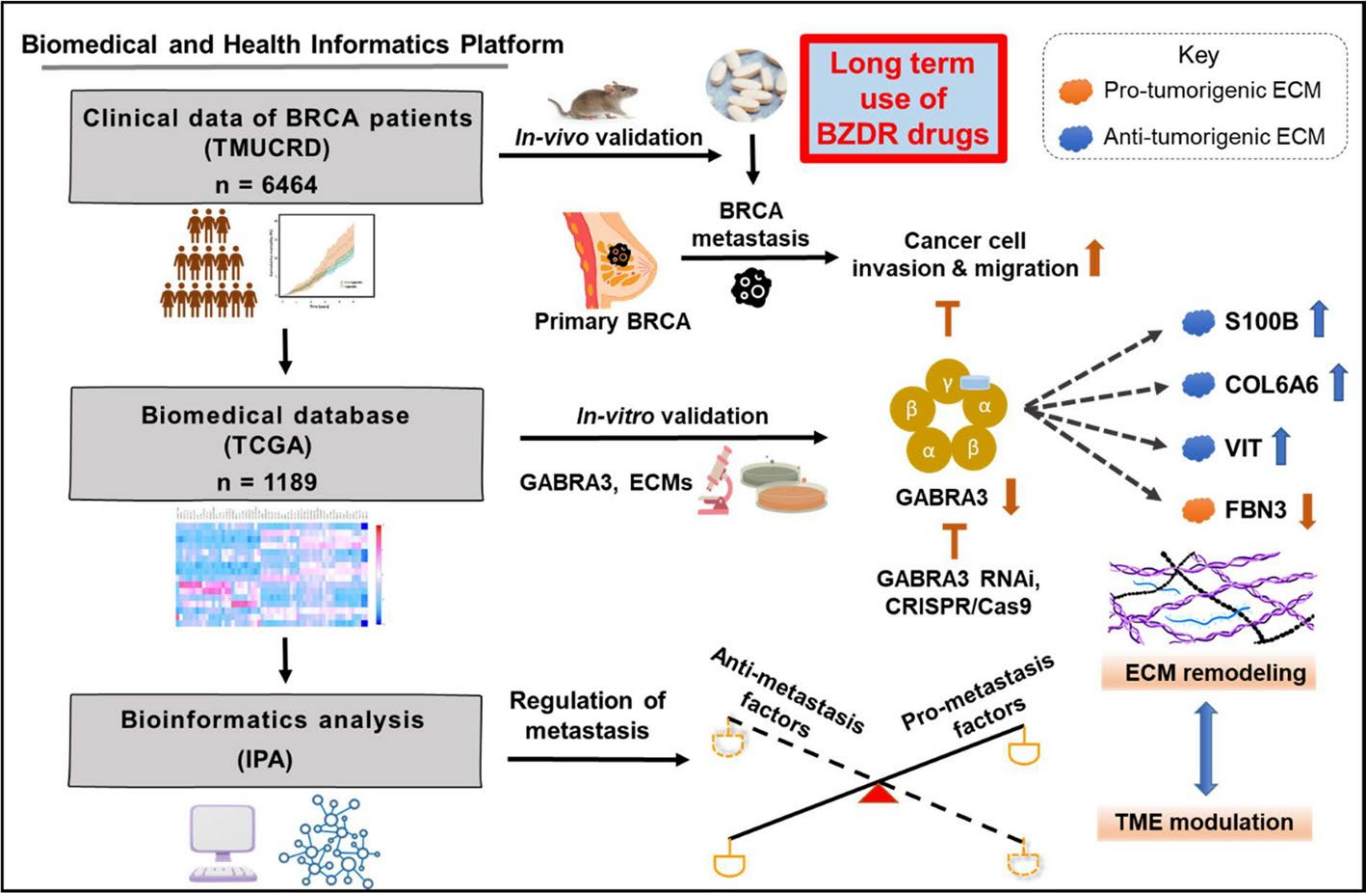

**Supplementary Information:**

The online version contains supplementary material available at 10.1186/s11658-025-00752-4.

## Introduction

Cancer is the second leading cause of death in the US and the leading fatality among people younger than 85 years. 2024 recorded 2,001,140 new cancer cases and projected 611,720 cancer deaths to occur in the US [1]. BRCA malignancy is a major cause of cancer-related death in women worldwide. In 2024, BRCA alone accounted for 310,720 new cases and 42,250 deaths in the US [1]. Hormone therapies for BRCA play a critical role in the significant decrease of BRCA mortality. However, side effects of hormone therapies include the loss of bone density, blood clots, and insulin resistance, amongst others [2]. Therefore, elucidating pivotal signaling, pro-/anti-tumorigenic factors and prognostic biomarkers involved in BRCA are essential and urgent for early diagnosis and development of anti-cancer therapeutics.

BZDRs are GABA-positive allosteric modulators widely used to treat anxiety, seizures, muscle spasm and panic disorders. BZDRs increase the frequency of chloride channel opening, which facilitates GABA receptor potential, and reduces neuronal firing [3]. Ion channel activities influence tumor metastasis [4–6]. GABA signaling is crucial to cell proliferation, migration, and differentiation [7–10]. BZDRs are typically recommended for short-term use due to risks of physical and psychological dependence. However, patients, including those diagnosed with cancers, are commonly prescribed BZDRs for prolonged use. Ironically, BZDRs have recently been patented as a potential anti-cancer drug to target MDM2-p53 interaction [11, 12], although its inhibitory binding activity is somewhat moderate [13]. Importantly, and contrary to the promotion of BZDR-usage, our earlier study showed by meta-analysis that usage of Diazepam (benzodiazepines, N05BA) and Zolpidem (related Z drugs, N05CF) is associated with increased risk of BRCA [14, 15]. Consistent results were also reported by others [16–18] on different types of cancer. Additionally, we have shown increases of cancers by 98% for brain, 25% for colorectal, and 10% for lung in BZDR users compared to cancer patients who are non-BZDR users [19]. Thus, we hypothesized that there are common BZDR-mediated molecular pathway(s) that contribute to carcinogenesis. Therefore, mapping the mechanisms of BZDR-mediated progression of BRCA, which is an important cancer model, is urgent especially since a significant 30% of BRCA patients are being prescribed BZDRs for long-term use.

Since BZDRs act via binding GABA type A receptors [20], we reasoned that profiling of GABA receptor expression will provide a lead on how GABA receptors modulate carcinogenesis [21]. Additionally, GABA/GABA receptors are known to modulate cancer metastasis via ECM-associated signaling [22]. This prompted us to investigate the underlying mechanisms of action of BZDRs in breast carcinogenesis. Here, we showed that BZDR drugs (Diazepam and Zolpidem) promote BRCA cell growth, migration and invasion. Retrospective analyses of clinical database from TMUCRD (Taipei Medical University Clinical Research Database) revealed an association of long-term use of BZDRs to reduced survival rate in BRCA patients (*p* = 0.034). Furthermore, based on retrospective studies we found that among 16 GABA receptors, GABRA3 was significantly upregulated during BRCA advancement, whereas GABRP was downregulated in the later stages of cancer. To further investigate the impact by BZDRs in breast carcinogenesis in vivo, we used mice model of human patient-derived xenograft whereby human BRCA cells were implanted through intratibial injection into immune-deficient mice [23, 24]. This intratibial model of studying metastasis is a recognised method for examining the later stages of MDA-MB231 cell-mediated metastasis [25–27]. Consistently, we showed that in vivo*,* BZDR treatment can promote BRCA MDA-MB231 cell-mediated metastasis in a xenograft mouse model. In vitro studies revealed that BZDRs increased the metastatic potential of BRCA cells, which can be significantly suppressed by both GABRA3-shRNA and GABRA3-CRISPR/Cas9. BZDRs downregulated anti-tumorigenic ECMs (S100B, COL6A6 and VIT) and upregulated pro-tumorigenic FBN3 in BRCA cells. Notably, knockout of GABRA3 repressed BZDR-induced carcinogenesis, probably influenced by a series of immunomodulators. Altogether, our clinical data analyses with molecular insights, suggest GABRA3-associated ECM signaling through the axes of GABRA3-S100B, GABRA3-COL6A6, GABRA3-VIT and/or GABRA3-FBN3. These networks might individually or collaboratively play pivotal roles in BZDR-triggered progression of BRCA, and these signaling networks could be targeted for therapeutic programs involving BZDR-treated cancer patients.

## Materials and methods

### TCGA database analysis

The TCGA breast carcinoma cohort (TCGA-BRCA) transcript dataset was downloaded from the R package, TCGA biolinks. All raw counts were normalized using DESeq2. In total, 1189 primary tissues were used in this study, including 1077 carcinoma tissues and 112 normal tissues. To determine whether specific genes showed statistically significant differential expression between normal and different tumor stages, a Student’s t-test was used. The *p* adjust value < 0.05 was used as threshold to define genes that showed statistically significant differential expression [28].

To gain insights into the cell and molecular mechanisms of action of BZDR in BRCA tumorigenesis, we next used various BRCA cell lines for in-depth analyses of potential signaling pathways and molecular interactions which might occur during BZDR-treatment.

### In vivo study of BZDR effects using MDA-MB231 cell-mediated metastasis in a mouse model

**Fig. 1:**
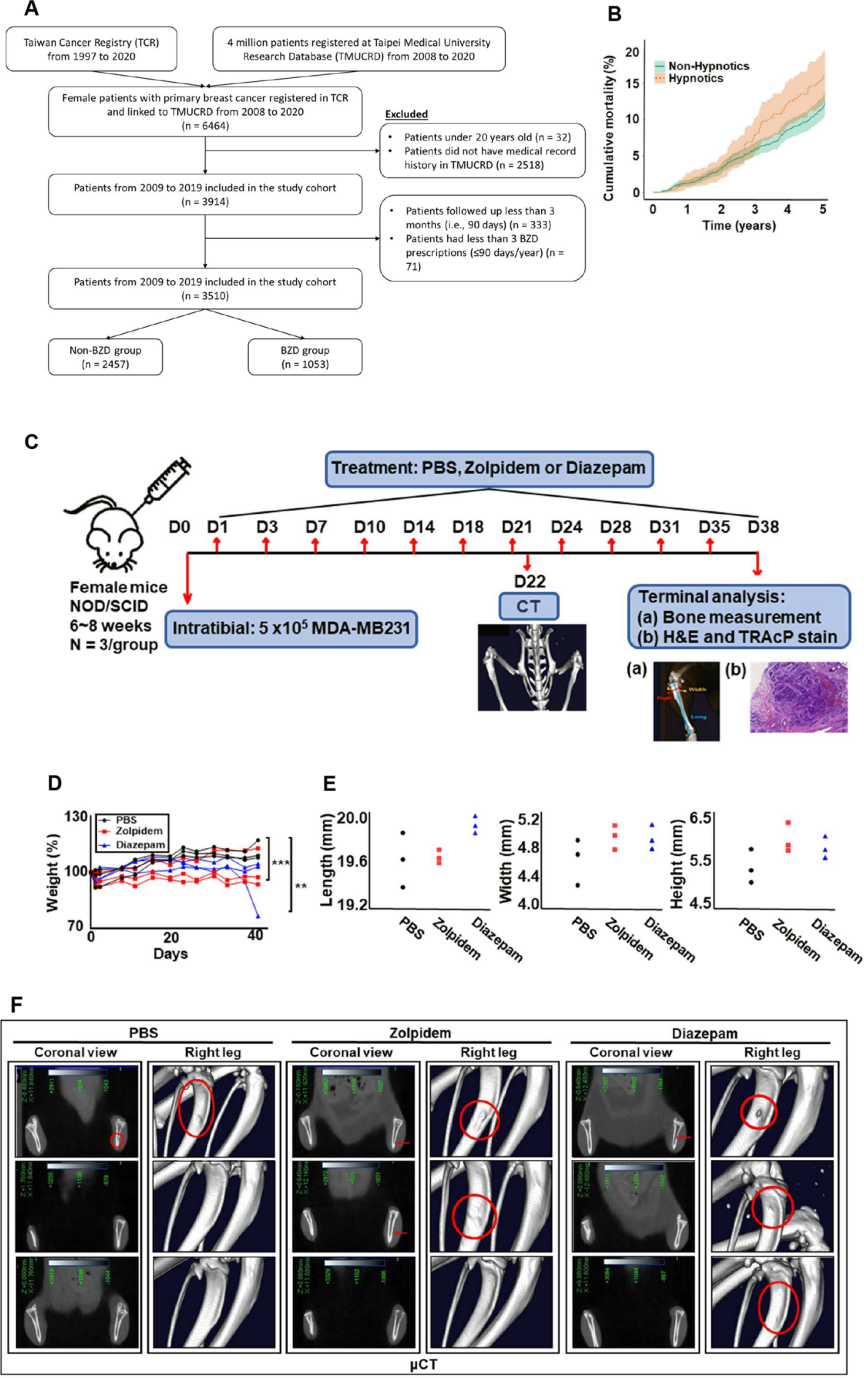

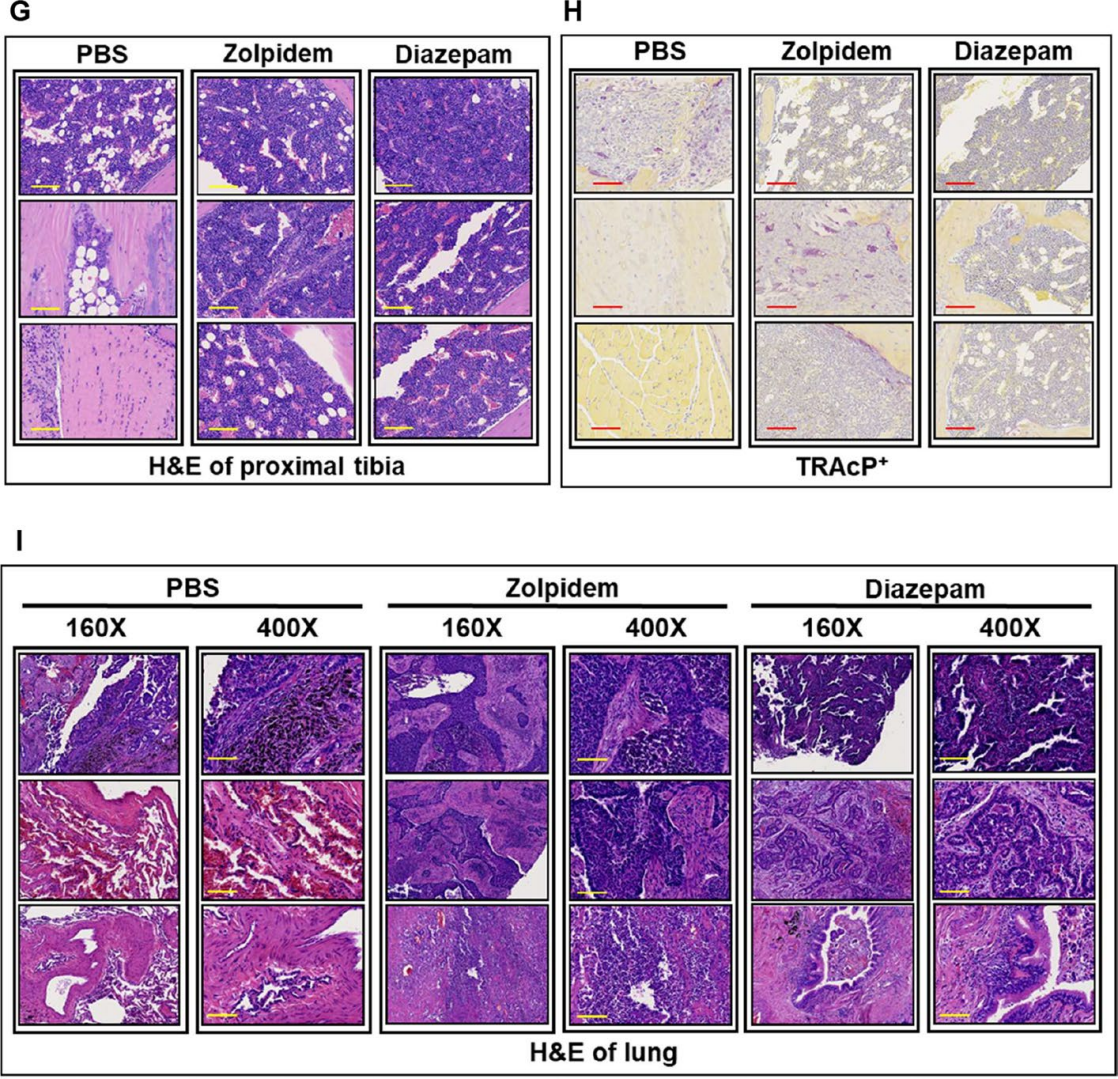
BZDR is associated with increased mortality in BRCA patients and BRCA metastasis in mice model. **A** Inclusion and exclusion criteria involved screening subjects obtained from TMUCRD. In total, 3510 patients are included in this study cohort, with n = 2457 in the non-BZDR group and n = 1053 in the BZDR group. **B** Cumulative mortality plots are graphically represented as in the patients’ survival analysis. Plots compared the mortality between users of Hypnotics vs. non-Hypnotics. The assessment of MDA-MB231 cell-mediated metastasis were carried out in vivo. **C** Experimental design of intratibial injection (details see Materials and Methods). **D** Measurements of body weight. The administration of PBS control and BZDRs at 10 mg/kg Zolpidem or 10 mg/kg Diazepam, were initiated one day after BRCA cell injection. The measurement of body weight was performed twice a week starting from the day after cell inoculation (Day 0). The percent change in body weight was calculated individually for each mouse based on the respective initial body weight. Compared to PBS control, reduced body weight for mice was observed upon treatment with Zolpidem (***p < 0.005) or Diazepam (**p < 0.01). **E** The extent of leg swelling, including width, length, and height which were measured by bone high-resolution microcomputed tomography (μCT). **F** In vivo μCT scans of proximal tibia. Red arrows and red circles indicate damaged area caused by cancer metastasis. **G** Histological assessment of tumor area using hematoxylin/eosin (H&E) staining of proximal tibia and **H** quantitation of tartrate-resistant acid phosphatase (TRAcP) + osteoclasts. **I** Metastatic assessment in lung were carried out using H&E staining. Scale bar is 100 μm, shown as yellow or red color line (—). **p < 0.01; ***p < 0.005

To support our clinical findings that BZDR promoted cancer advancement in BRCA patients, we studied the effects of BZDR treatment, in vivo, in NOD/SCID mice induced with an osteolytic BRCA cell line, MDA-MB231, and monitored the impact of BZDR on BRCA progression. The tibial injection model was chosen in our study to focus our investigation on the importance of BZDRs in mediating tumor cell interactions with the tissue microenvironment [25–27]. Six to eight-week-old female NOD.CB17-*Prkdc*^*scid*^/NcrCrl mice (Charles River, UK) were used to evaluate in vivo drug efficacy of orally administered BZDRs in a MDA-MB231 cell-mediated metastasis model. On Day 0, 5 × 10^5^ MDA-MB231 BRCA cells were injected into the right tibia of the mice using a 27G needle, with an injection volume of 25 μL [25, 29]. The administration of control (PBS) and BZDRs, including 10 mg/kg Zolpidem or 10 mg/kg Diazepam, were initiated one day after BRCA cell injection (see schedule presented in Fig. 1). The control/BDRs were administered via oral gavage three times per week. The measurement of body weight was performed twice a week starting from the day after cell inoculation (Day 0). The percent change in body weight was calculated individually for each mouse based on the respective initial body weight. The BRCA cell-induced damage to the proximal tibia of each animal was assessed on Day 22 using in vivo micro-CT imaging (Quantum FX micro-CT, Perkin Elmer Co.). The imaging parameters included a tube potential of 90 kVp and a tube current of 160 μA. Tomographic images were acquired using the Quantum FX micro-CT viewer (Perkin Elmer Co.) to visualize bone erosion. The day after the final administration of BZDRs, experimental animals were sacrificed using CO_2_ euthanasia. Images of the legs were taken, and the extent of leg swelling were measured, including width, length, and height. Leg bones and lung tissues were harvested and preserved in 10% formaldehyde for subsequent H&E (hematoxylin and eosin) and TRAcP staining. All in vivo experiments were performed in accordance to the “Guide for the Care and Use of Laboratory Animals” (NIH publication 86-23, revised 1985) and was approved by the Committee on the Ethics of Animal Experiments of TRINEO BIOTECHNOLOGY CO., LTD. (IACUC-2024-SH-018) [30].

Source of patient data from TMUCRD; Clinical population examined from TMUCRD; BZDR classification and exposure**;** Cell lines & reagents; qRT-PCR; shRNA of GABRA3; CRISPR/Cas9 system; Cell growth assay; Cell migration assay; Cell invasion assay; IPA analysis; Statistical analysis, are described in the Supplementary Information. All human cell lines including MCF7 (RRID:CVCL_0031), MDA-MB231 (RRID:CVCL_0062) and MCF10A (RRID:CVCL_0598), used in this work are tested to be mycoplasma-free cells and are authenticated using STR profiling within the last three years.

### Statistical analysis

A comparison of cumulative probabilities in competing for the risk of death was estimated using modified Kaplan–Meier and Gray methods. We tested differences in the time to event between patients in the BZDR-user and non–BZDR–user groups using a log-rank test. Details are in the Supplementary information. Briefly, statistical tests were 2-sided, and a *P*-value < 0.05 was considered to indicate statistical significance.

## Results

### BZDR is associated with increased mortality in BRCA patients and supported by metastasis in mice model

To evaluate the pathophysiological relevance of BZDR usage in cancer patients, we analyzed clinical data from the TMUCRD, which has gathered comprehensive medical records from three medical centers located in Taiwan, including TMUH, WFH, and SHH. Retrospective analyses of the clinical data from TMUCRD (n = 3510 BRCA patients) revealed n = 2457 non-BZDR users and n = 1053 BZDR users (Fig. 1A; see details in Materials and Methods section).

Cumulative mortality plotter analysis revealed that long-term use of hypnotics, including Estazolam (benzodiazepine derivatives, N05CD), Zolpidem (related Z-drugs, N05CF) and Zopiclone (related Z-drugs, N05CF), was associated with increased mortality rate in BRCA patients (HR (95% CI) = 1.34 (1.02–1.76); *p*-value = 0.034) (Fig. 1B) [31]. Specifically, the 5-year cumulative mortality probabilities in competing for the risk of death was 15.6% for users vs. 11.7% for non-users of hypnotics among BRCA patients. In sum, we observed that long-term BZDR usage is associated with poor survival rate amongst BRCA patients. Since a significant 30% of BRCA patients are still prescribed BZDRs for prolonged usage, it is urgent to investigate the underlying molecular mechanisms of BZDR treatment to reveal which pathway(s) were involved and how to intervene the BRCA progression candidates.

To demonstrate the effects of BZDRs in BRCA development in vivo, we inoculated NOD/SCID mice with the osteolytic BRCA cell line MDA-MB231 (Fig. 1C). Intratibial injection was performed with the right tibia of the mice (for details, see Materials and Methods). Tested materials include PBS (as control), 10 mg/kg Zolpidem and 10 mg/kg Diazepam, which were administrated via oral gavage three times per week. Figure 1D show reduced body weight for mice treated with BZDR compared to PBS control (** *p* < 0.01; *** *p* < 0.005). The percent change in body weight was calculated individually for each mouse based on the respective initial body weight. The leg swelling status measured by width, length and height are shown in Fig. 1E. Additionally, in vivo μCT revealed images of damaged proximal tibia (Fig. 1F). White and gray images represent the areas with relatively high bone density. Red arrows and circles indicate bone erosion observed mainly in BZDR-treatment. This observation was verified by histology, H&E and TRAcP staining (Fig. 1G and H). Notably, BZDR-treatment showed clear metastasis to the lung and localization in the tibia (for bone microenvironment interactions and TME maintenance) (Fig. 1I) [25–27] supporting that BZDRs promote cancer advancement in vivo.

### Diazepam and Zolpidem induce BRCA cell migration

**Fig. 2:**
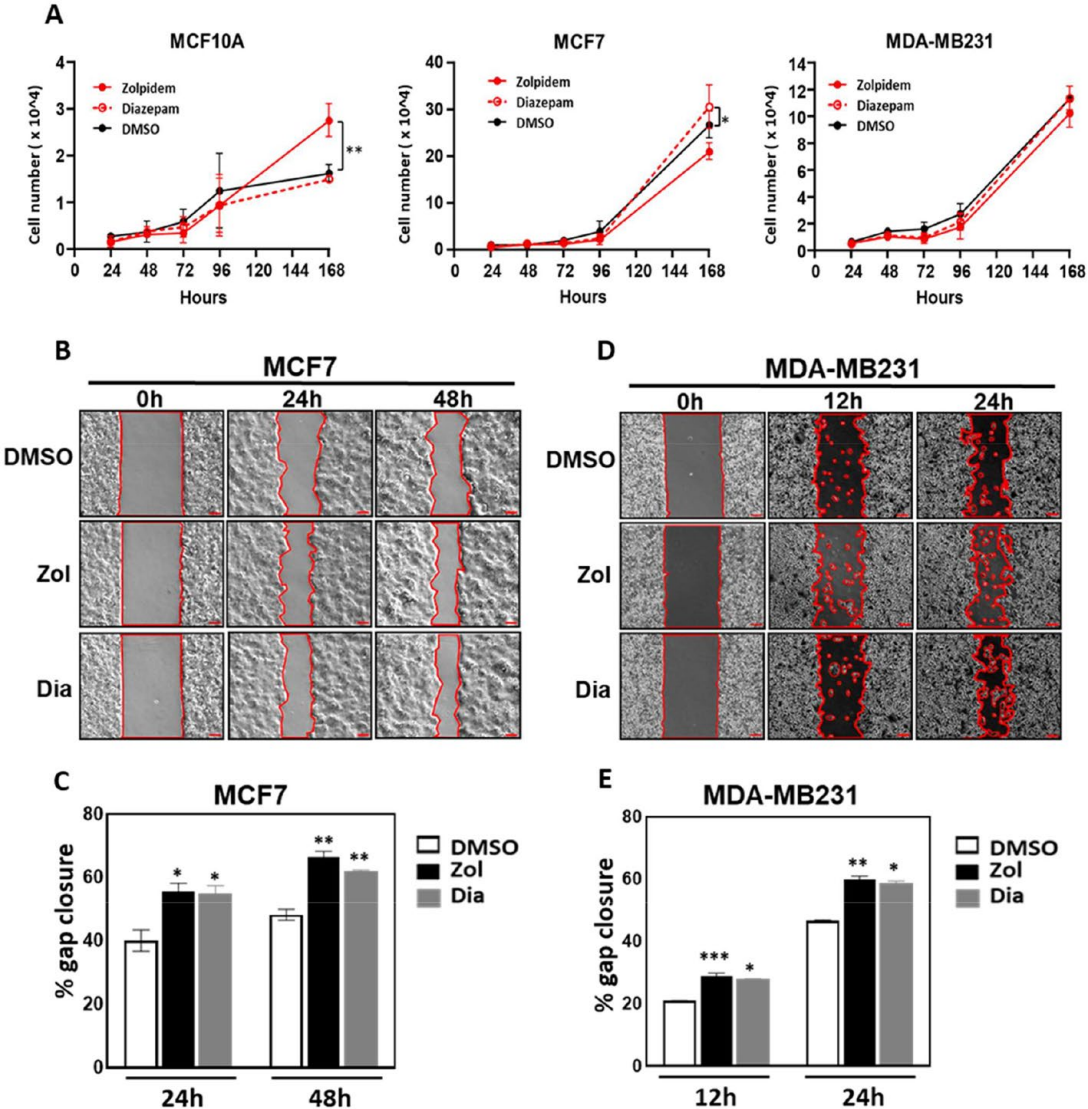
Treatment of BRCA cell lines with Diazepam and Zolpidem induced cell migration. The effects of two BZDR drugs (benzodiazepines and related Z-drugs), Zolpidem and Diazepam, on BRCA cell growth was determined by: **A** Trypan Blue exclusion test to study the cell growth of a non-carcinoma cell line (MCF10A), a low metastatic cell line (MCF7) and a highly metastatic cell line (MDA-MB231). Zolpidem significantly increased cell growth in MCF10A, whereas Diazepam stimulated MCF7 cell growth significantly (*p < 0.05; **p < 0.01). Image and quantitative analyses of cell migration assay were carried out for **B**, **C** MCF7 and **D**, **E** MDA-MB231 cells. Compared with DMSO control, treatment with Zolpidem and Diazepam significantly increased BRCA cell migration. The migration rate (% gap closure) was quantitatively normalized to the corresponding 0 h time point controls. Data are representative of means ± SD (n = 3). *p < 0.05; **p < 0.01; ***p < 0.005

Since BZDRs are associated with heightened risk of BRCA [14], especially with the increased mortality in BRCA patients on long term treatment with BZDR (Fig. 1), it was pertinent for us to gain insights into the cell and molecular effects of Diazepam and Zolpidem in BRCA progression. To this end, we used two metastatic BRCA cell lines, MCF7 and MDA-MB231, and a non-carcinoma cell line, MCF10A, for comparison (Fig. 2A). We demonstrated significant increase in cell growth for MCF10A and MCF7 after 7-day treatments with Zolpidem and Diazepam (* *p* < 0.05; ** *p* < 0.01). Additionally, there were significant increases in cell migration in MCF7 (20% increase) (Fig. 2B, C, * *p* < 0.05; ** *p* < 0.01) and in MDA-MB231 (15% increase) (Fig. 2D, E, * *p* < 0.05; ** *p* < 0.01; *** *p* < 0.005). These results indicate that BZDRs were associated with metastatic progression of BRCA.

BZDRs are known to exert anxiolytic and anticonvulsant effects via central-type GABA type A receptors [32]. Increasing evidence suggest that GABA receptors play a key role in cancer development and progression, including BRCA [33, 34]. GABA/GABA receptor-signaling may contribute to the maintenance of a tumor microenvironment (TME), for example, in mediating cytokine production by immune cells [3]. Nevertheless, there remains many questions, like how GABA receptor mediates crosstalk amongst non-cancer cells, immune cells and tumor cells in the TME. Thus, we further sought to clarify the expression profile of key GABA receptors to gain insights into how BZDRs mediate breast carcinogenesis and advance the disease.

### BZDRs regulate the expression of GABRA3 and GABRP in breast carcinogenesis

**Fig. 3:**
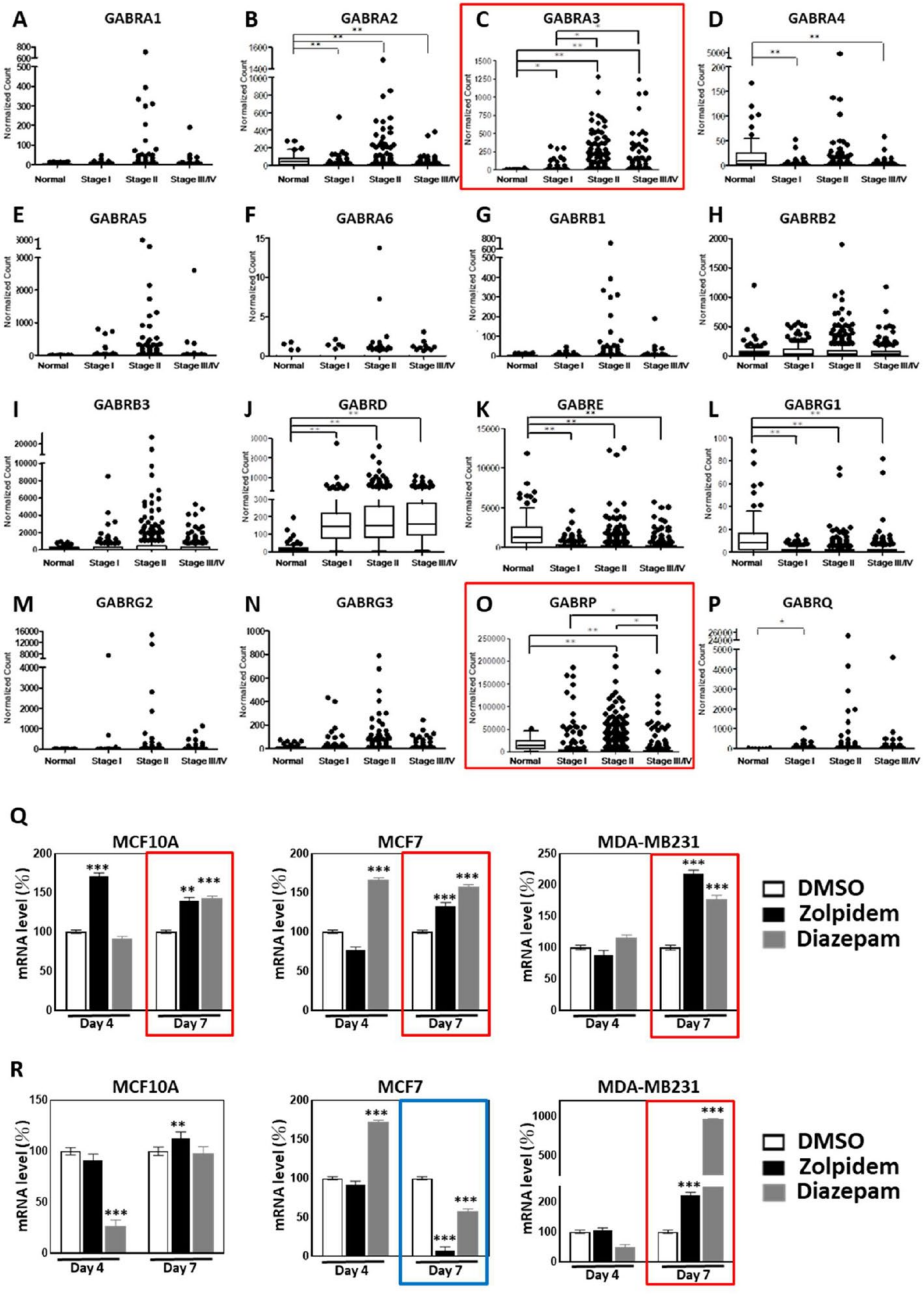
BZDR drugs significantly upregulated GABRA3 expression which is associated with BRCA staging. To characterize the expression profiles of GABA receptors in human breast cancer, we analyzed The Cancer Genome Atlas (TCGA) database (details in Materials and Methods). In total, 1189 primary samples were examined, including 112 normal breast tissues and 1077 carcinoma breast tissues (Stage I: 182, Stage II: 624, Stage III: 251 and Stage IV: 20). 16 GABA receptors were identified, including **A** GABRA1, **B** GABRA2, **C** GABRA3, **D** GABRA4, **E** GABRA5, **F** GABRA6, **G** GABRB1, **H** GABRB2, **I** GABRB3, **J** GABRD, **K** GABRE, **L** GABRG1, **M** GABRG2, **N** GABRG3, **O** GABRP and **P** GABRQ. Additionally, log scale plots were provided for clearer presentation of the findings (Supplementary Figure S1). Gene expression of GABRA3 and GABRD was upregulated in carcinoma tissues, compared to normal tissues throughout stagings (**C**, **J**). In contrast, GABRA2, GABRE, GABRG1 and GABRP were down-regulated in breast carcinoma, compared to normal controls (**B**, **K**, **L**, **O**). The expression of GABRA3 and GABRP (red boxes; panels **C** and **O**, respectively) appeared to be associated with staging events. In advanced status of the disease (stage III/IV), GABRA3 was significantly over-expressed compared to early stage I (**C**). However, GABRP was further reduced in stage III/IV of BRCA, compared to early stage (stage I) (**O**). The expression of GABRA3 and GABRP in vitro in the 3 BRCA cell lines, in response to BZDR stimuli are shown in (**Q**) and (**R**), respectively. Treatment of BRCA cell lines with Zolpidem and Diazepam for 7 days up-regulated the expression of pro-tumorigenic factors, GABRA3 in MCF10A, in MCF7 and MDA-MB231 cells (**Q**, red boxes) and GABRP in MDA-MB231 cells (**R**, red box). GABRP is reduced upon BZDR treatment (7-Days) in MCF7 (**R**, blue box). *p < 0.05; **p < 0.01; ***p < 0.005

To clarify the pathophysiological relevance of GABA receptors in BRCA development, we studied the expression profiles of GABA receptors in BRCA patient tissues. Sixteen GABA receptors: GABRA1, GABRA2, GABRA3, GABRA4, GABRA5, GABRA6, GABRB1, GABRB2, GABRB3, GABRD, GABRE, GABRG1, GABRG2, GABRG3, GABRP and GABRQ were examined by TCGA database analysis (Fig. 3A–P), out of which 2 receptors, GABRA3 and GABRD (Fig. 3C, J), emerged with significant upregulation in breast carcinoma tissues compared to corresponding receptors in normal breast tissues (* *p* < 0.05; ** *p* < 0.01). By contrast, 4 other GABA receptors (GABRA2, GABRE, GABRG1 and GABRP) were significantly downregulated in BRCA tissues (* *p* < 0.05; ** *p* < 0.01). Throughout cancer staging, TCGA database showed consistent reduction in expression levels of GABRA2, GABRE, GABRG1 and GABRP by up to 32.7%, 18.7%, 15.4% and 5.9%, respectively (Fig. 3B, K, L, O). Additionally, log scale plots were provided for clearer presentation of the findings (Supplementary Figure S1). Details of the expression levels of these GABA receptors are shown in the Supplementary Information (Supplementary Table S1). Although data derived from TCGA database provide helpful pointers for empirical analysis, the potential bias with TCGA data for cancer research may occur; recently, Liu et al., and Liu et al., suggested [35, 36] that TCGA data analyses often rely on highly-expressed genes as indicators of carcinogenesis. Technical bias in TCGA may also be caused by cross-hybridization and PCR amplification bias. Furthermore, biological bias may arise from tumor heterogeneity and sample purity. Hence, recognizing and addressing these biases is crucial for caution in data interpretations to achieve more realistic and accurate cancer research and clinical applications.

Notably, among 6 of the above-identified GABA receptors, GABRA3 and GABRP are associated with staging events of breast carcinogenesis (Fig. 3C and O, respectively). While GABRA3 was significantly upregulated with the advancement of BRCA, GABRP was downregulated in the later stage (red boxes, * *p* < 0.05; ** *p* < 0.01). These results indicate that both receptors are likely involved in BRCA initiation and progression. Consistent with previous studies [32], our dynamic expression profiling of GABA receptors reflects the potential modulation of carcinogenesis. Henceforth, to investigate the underlying mechanisms of BZDR-induced advancement of BRCA, we focused our attention on the regulation of GABRA3 and GABRP in BRCA patient tissues (Fig. 3C and O). Thus, we further examined the expression profiles of GABRA3 and GABRP in BRCA cells treated with Diazepam and Zolpidem. We found that a 7-day treatment regime with these BZDRs (regarded as prolonged treatment under in vitro condition), significantly upregulated GABRA3 in both non-carcinoma MCF10A and carcinoma cells, MCF7 and MDA-MB231 (Fig. 3Q, red boxes). GABRA3 plays a pro-tumorigenic role [9] and enhances the metastatic potential of BRCA. Combined with our observation that GABRA3 was significantly upregulated upon BZDR stimulation (Fig. 3Q), we propose that Zolpidem and Diazepam likely promote BRCA advancement and metastatic progression by stimulating the GABRA3 pathway. A 7-day treatment with Zolpidem and Diazepam significantly increased GABRP expression in the highly metastatic MDA-MB231 cells (Fig. 3R, red box), but a reduction in the low metastatic MCF7 cells (Fig. 3R, blue box). This is consistent with a previous study in which GABRP was recognized as a potent activator of triple negative breast cancer (TNBC) [37]. Altogether, our results indicate GABRA3 to play key roles in BZDR-mediated BRCA progression.

The ECM proteins are known to be involved in GABA receptor-associated cancer development and progression [22, 38]. Therefore, we next defined the potential interplay between GABA receptors and ECM molecules in BZDR-treatment. This is essential for future intervention of cancer metastasis and development of anti-cancer therapeutics for BRCA patients under BZDR treatment.

### Carcinogenesis-associated ECM molecules respond to BZDR treatment to exacerbate BRCA

**Fig. 4:**
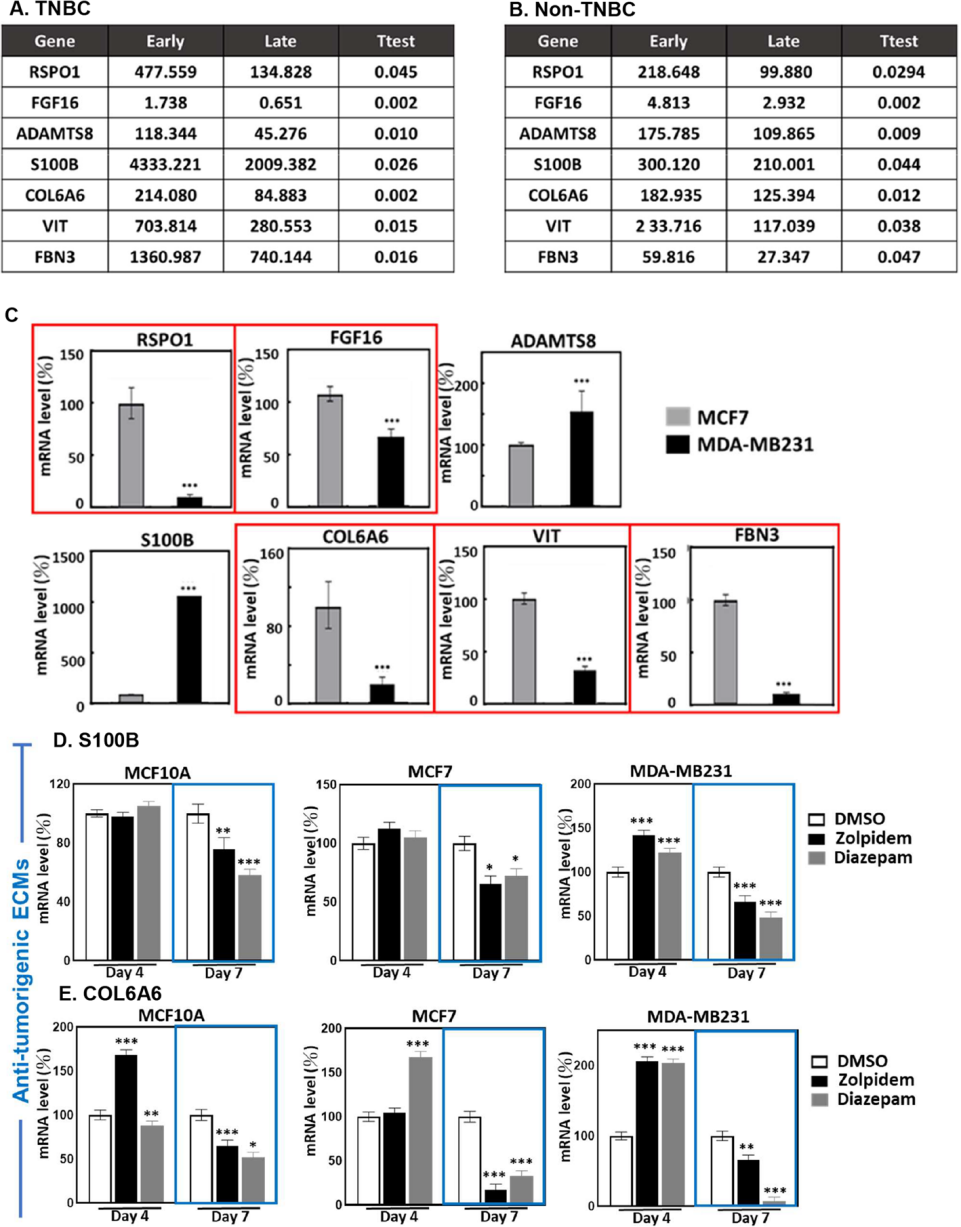

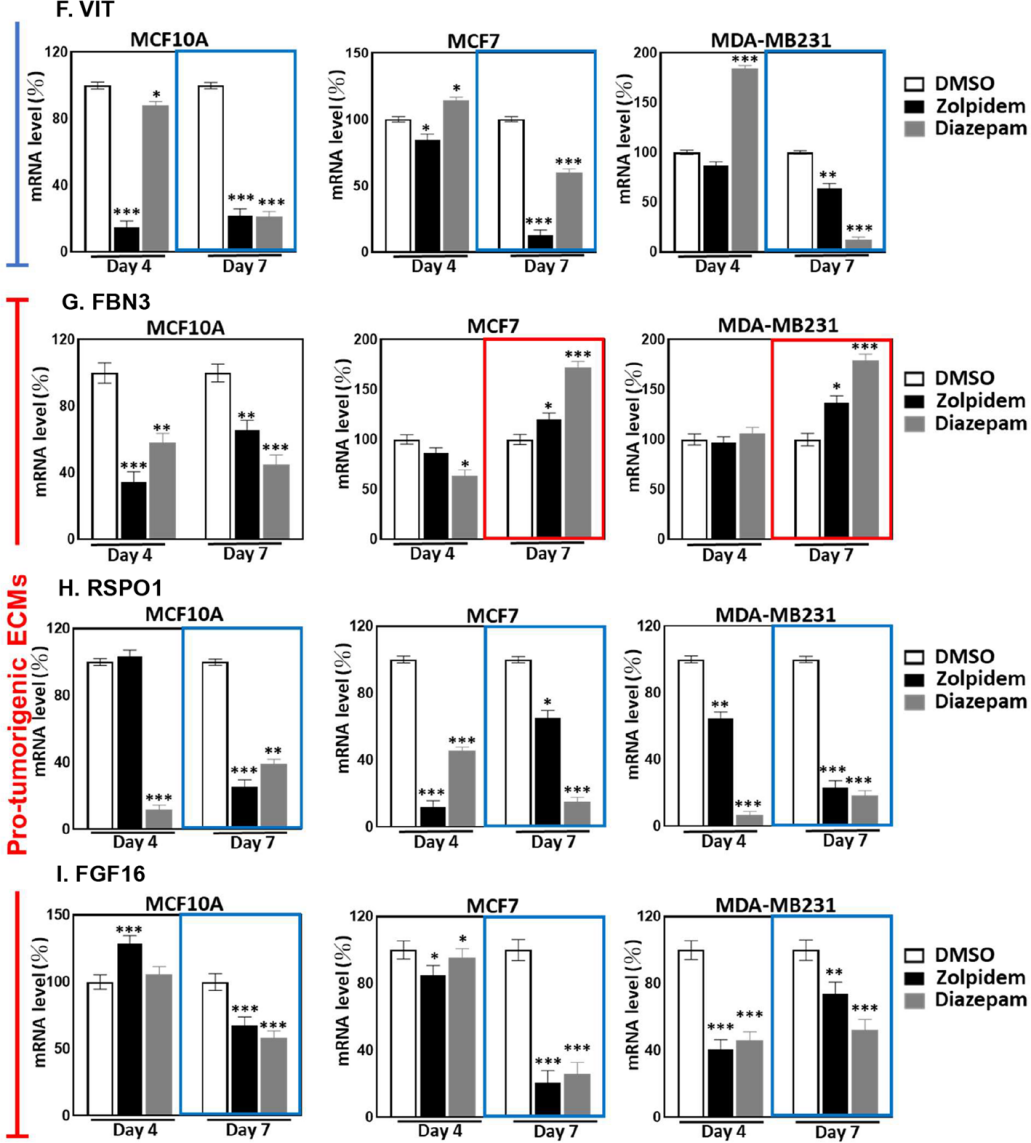
BRCA cells treated with BZDR show dynamic expression of carcinogenesis-associated ECMs. To identify the potential ECM molecules which may be involved in BRCA development and/or progression, we analyzed TCGA database based on our earlier findings [41]. In total, 1516 ECM molecules were studied in **A** Triple negative BRCA (TNBC) and **B** non-Triple negative (non-TNBC) BRCA (including HER2, LumA, LumB, normal-like). We showed that 7 ECMs: RSPO1, FGF16, ADAMTS8, S100B, COL6A6, VIT and FBN3, were significantly down-regulated in later stages II, III, IV of BRCA, compared to early stage I of the disease. In **A**, 187 primary samples were examined, including 25 stage I, 132 stage II, 27 stage III and 3 stage IV. In **B**, 878 primary samples were examined, including 157 stage I, 484 stage II, 221 stage III and 16 stage IV. The expression patterns of these ECMs were affirmed in MCF7 and MDA-MB231 cells (**C**). A consistent trend (high expression in MCF7 and low expression in MDA-MB231 cells) was found in vitro with RSPO1, FGF16, COL6A6, VIT and FBN3 (red boxes). Expression profiles of key ECM molecules were determined upon BZDR treatment, which included **D** S100B, **E** COL6A6, **F** VIT, **G** FBN3, **H** RSPO1and **I** FGFG16. Zolpidem and Diazepam were found to have significantly suppressed anti-tumorigenic ECMs (S100B, COL6A6 and VIT) (blue boxes), and activated pro-tumorigenic FBN3 (red boxes) in BRCA cells. *p < 0.05; **p < 0.01; ***p < 0.005

The ECM constitutes a highly dynamic network, and ECM remodeling may promote a cellular microenvironment leading to cancer spread [39, 40]. We recently identified 1516 ECM molecules associated with cancer development [41]. To better understand how carcinogenesis-associated ECMs may participate in BZDR-promoted BRCA advancement, we analyzed these 1516 ECMs in the TCGA database and examined 1067 primary tissues, including 189 Triple negative breast cancer (TNBC) and 878 non-TNBC (including HER2, LumA, LumB, normal-like). From amongst 1516 ECMs examined, seven (RSPO1, FGF16, ADAMTS8, S100B, COL6A6, VIT/vitrin and FBN3) showed dramatic reduction in BRCA stages II, III and IV (Fig. 4A, B). This is consistent in both TNBC and non-TNBC contents, these 7 ECMs may be key correlates with BRCA progression in patients on prolonged treatment with BZDRs. We next used qRT-PCR to quantify and validate the gene expression in BRCA cells (Fig. 4C). Our results indicated that out of these 7 ECMs studied, 5 ECMs (RSPO1, FGF16, COL6A6, VIT and FBN3) showed gene expression trend that was consistent with that of the TCGA database. These 5 ECMs were significantly downregulated in highly-invasive MDA-MB231 cells, compared to the less-invasive MCF7 cells (Fig. 4C, red boxes). MDA-MB231 cells are more aggressive than MCF7 cells and are generally considered as later stages of BRCA model. Nevertheless, we should not exclude the possibility that the dynamic expression of the examined ECMs may also be attributed to other factors, for example the ER/PR status.

To better understand how the identified ECMs might be involved in the interaction/signaling axis of BZDR-GABA receptors, we examined the expression profiles of the ECMs in BZDR-treated cells. Out of 7 ECMs, six (S100B, COL6A6, VIT, FBN3, RSPO1 and FGF16) showed dynamic expression upon BZDR-treatment (Fig. 4D–I), supporting that these ECMs are intensively involved in BZDR-treatment and BZDR-mediated signaling. We observed that a 7-day treatment with BZDRs downregulated anti-tumorigenic ECMs, S100B, COL6A6 and VIT, in both non-carcinoma (MCF10A) and carcinoma cells (MCF7 and MDA-MB231) (Fig. 4D–F) (blue boxes, * *p* < 0.05; ** *p* < 0.01; *** *p* < 0.005). Consistently, S100B and VIT, which are anti-tumorigenic ECMs, were downregulated in primary BRCA tissues, compared to normal tissue counterparts [42, 43]. Furthermore, low expression level of COL6A6 (another anti-tumorigenic ECM) was significantly associated with advanced pathological stage and large tumor size [44]. Altogether, our results reinforce that downregulation of anti-tumorigenic S100B, VIT and COL6A6 potently advances BRCA, and that these anti-tumorigenic ECMs are repressed by BZDRs. Consistently, FBN3, a pro-tumorigenic prognostic marker of metastasis of BRCA to bone [45], was significantly upregulated in both MCF7 and MDA-MB231 cells upon treatments with BZDR for 7 days (Fig. 4G, red boxes). On the other hand, pro-tumorigenic factors, RSPO1 and FGF16, were significantly downregulated in MDA-MB231 by BZDR (Fig. 4H, I). The expression levels of ADAMTS8 remained unchanged during BZDR-treatment (data not shown), indicating that this anti-tumorigenic ECM molecule is uninvolved in BZDR stimulation [46]. Altogether, our experimental data highlight the complexity of BZDR-induced molecular networks in BRCA. BZDRs appear to effectively and consistently regulate the expression of ECM molecules that are associated with BRCA progression. Future studies using RNAi of each and/or combinations of these ECM candidates would help to corroborate the involvement of ECMs in BZDR-mediated signaling pathways.

### BZDRs provoke crosstalk between GABRA3 and ECM molecules to promote BRCA advancement

**Fig. 5:**
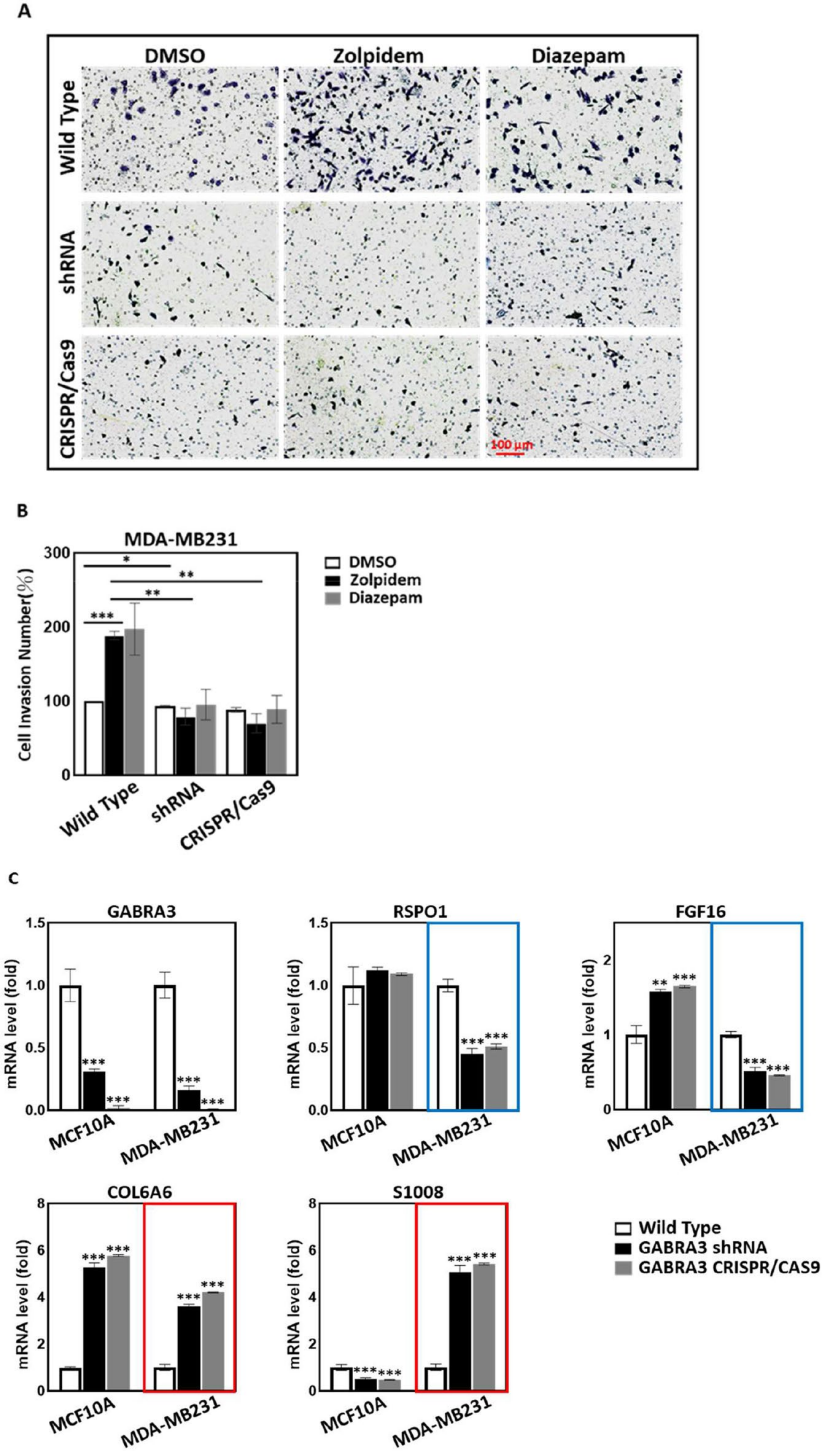

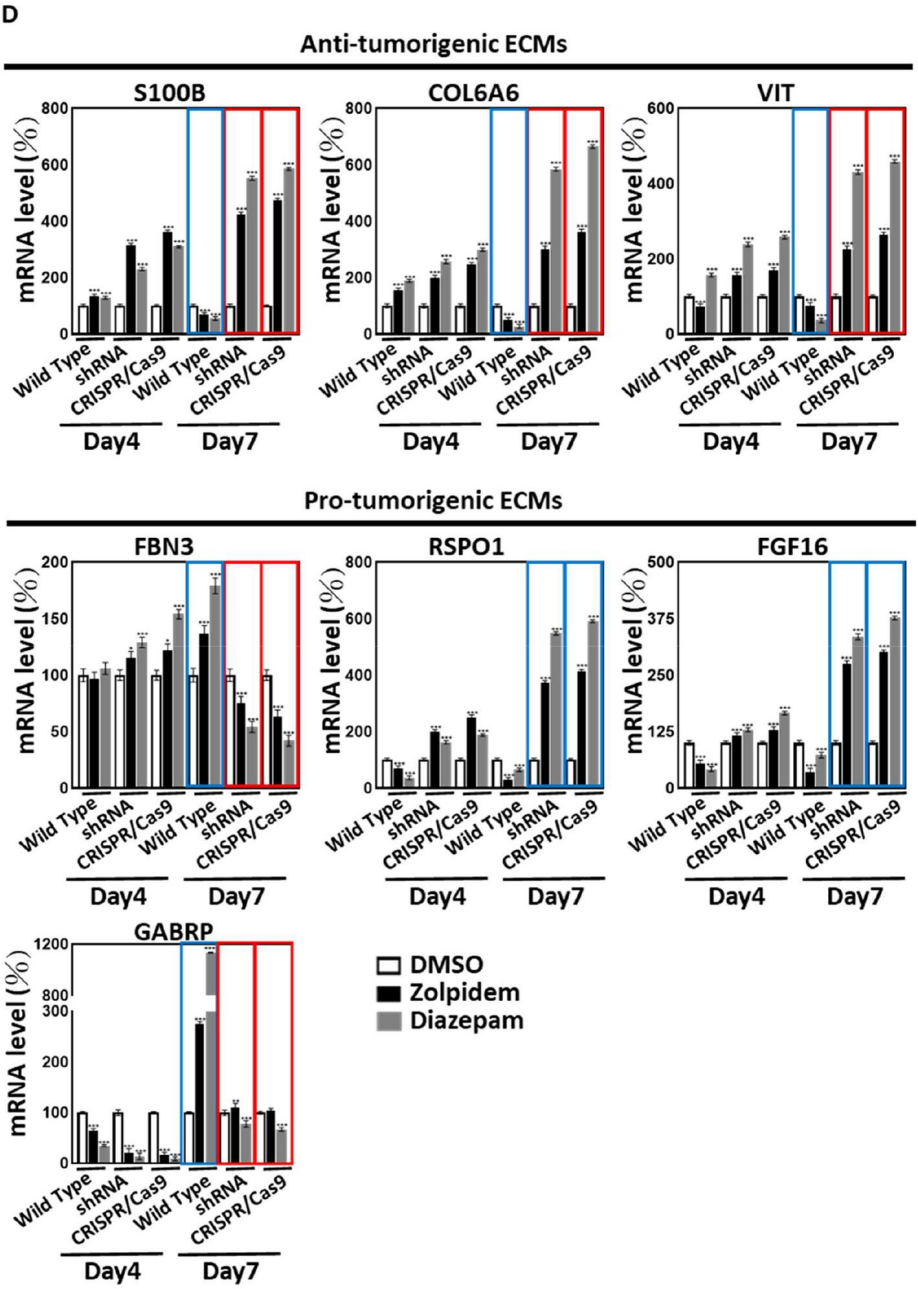
BRCA cells treated with BZDRs display exacerbated cancer progression via networks of GABRA3 and ECMs. To investigate whether and how BZDR may mediate breast carcinogenesis, cell invasion assays were performed (**A**) and quantified (**B**). Both BZDRs, Zolpidem and Diazepam, increased cell invasiveness of MDA-MB231 cells (*p < 0.05; ***p < 0.005). Of note, both GABRA3-shRNA and GABRA3-CRISPR/Cas9 significantly reduced cell invasion induced by BZDR-treatment (**p < 0.01). **C** Shows the endogenous levels of key ECMs in response to GABRA3 knockdown or knockout in MCF10A and MDA-MB231 cells. Effective GABRA3-shRNA and GABRA3-CRISPR/Cas9 was affirmed by qRT-PCR (**C**). Blue boxes show that without BZDR stimulation, GABRA3 knockdown caused reduction of pro-tumorigenic RSPO1 and FGF16 in MDA-MB231. Red boxes show that GABRA3-shRNA and GABRA3-CRISPR/Cas9 led to upregulation of anti-tumorigenic COL6A6 and S100B in MDA-MB231. **D** GABRA3-shRNA and GABRA3-CRISPR/Cas9 cells were treated with BZDR dugs, and the mRNA expression of GABRP and key ECMs (RSPO1, FGF16, S100B, COL6A6, VIT and FBN3) was quantified by qRT-PCR. 7-day treatment with BZDRs significantly reduced the expression of anti-tumorigenic ECMs (S100B, COL6A6 and VIT) (blue boxes). BZDRs upregulated expression of FBN3 (a prognostic marker of BRCA patients). In contrast, BZDRs also decreased the levels of pro-tumorigenic RSPO1 and FGF16. Of note, GABRA3-shRNA and GABRA3-CRISPR/Cas9 significantly restored effects caused by BZDRs in S100B, COL6A6, VIT, FBN3 (red boxes), as well as RSPO1 and FGF16. These results indicate the comprehensive networks between BZDR-associated ECMs and GABRA3 in breast carcinogenesis. GABRP is upregulated by BZDR treatment (7-Day treatment, red box), which was suppressed by GABRA3-shRNA and GABRA3-CRISPR/Cas9 (blue box). *p < 0.05; **p < 0.01; ***p < 0.005

Studies have shown that BZDRs exert their neuro-therapeutic effects through interaction with the GABA type A receptor [20]. As a pro-tumorigenic receptor, GABRA3 has been reported to stimulate BRCA metastasis [9]. On this premise, we further revealed that both of the BZDRs, Zolpidem and Diazepam, significantly increased cancer cell invasion ability by up to twofold with the highly metastatic MDA-MB231 cells (Fig. 5A, B). Altogether, our clinical findings and experimental results from both in vivo mice studies and in vitro cell studies indicate advancement of BRCA disease, concordant with increased mortality in BRCA patients on long-term use of BZDRs. To avoid misinterpretation due to potential off-target effects, we specifically knocked down and also knocked out GABRA3 gene using shRNA and CRISPR/Cas9 approaches, respectively. Both GABRA3-shRNA and GABRA3-CRISPR/Cas9 in MDA-MB231 cells consistently reduced cell invasion caused by BZDRs (Fig. 5A, 5B) (* *p* < 0.05; ** *p* < 0.01; *** *p* < 0.005), confirming that GABRA3 plays a pivotal role in promoting cancer progression in BZDR-mediated cancer signaling, during BRCA progression. We next examined the correlation between GABRA3 and the previously identified ECM molecules [41], based on hints that ECMs are involved in cancer progression (see Fig. 4A–C). GABRA3 knockdown using shRNA effectively reduced its gene expression by 70% and 85% in MCF10A and MDA-MB231 cells, respectively (Fig. 5C). In CRISPR/Cas9-mediated GABRA3 knockout cells, qRT-PCR analysis indicated minimal expression of GABRA3 gene.

Without BZDR stimulation and with suppression of GABRA3, we showed significant downregulation of pro-tumorigenic factors, RSPO1 and FGF16 in MDA-MB231 cells (Fig. 5C, blue boxes) and upregulation of anti-tumorigenic factors, COL6A6 and S100B in MDA-MB231 cells (Fig. 5C, red boxes). The above observations are consistent in both shRNA and CRISPR/Cas9 systems. These results indicated that the pro-tumorigenic role of GABRA3 might be exacerbated by its collaboration with the pro-tumorigenic ECM networks in breast carcinogenesis. To affirm the involvement of ECMs triggered by BZDR stimulation, we further treated MDA-MB231 cells that have been knocked down by GABRA3-shRNA and knocked out by GABRA3-CRISPR/Cas9 with BZDRs. We found that BZDRs suppressed anti-tumorigenic S100B, COL6A6 and VIT [47–49] (Fig. 5D, blue boxes) in the presence of GABRA3. Importantly, the BZDR-mediated reduction of the anti-tumorigenic ECMs: S100B, COL6A6 and VIT were restored by GABRA3-shRNA and GABRA3-CRISPR/Cas9 (Fig. 5D, red boxes). Taken together, we reasoned that the dynamic axes of GABRA3-S100B, GABRA3-COL6A6, GABRA3-VIT play pivotal roles in BZDR-mediated BRCA progression. Consistently, upregulation of FBN3 (a prognostic marker of BRCA patients) caused by BZDRs (Fig. 5D, red box) was suppressed by GABRA3-shRNA and GABRA3-CRISPR/Cas9 (Fig. 5D, blue box). These results collectively corroborate that BZDRs induce BRCA advancement via GABRA3-associated ECM signaling, through the axes of GABRA3-S100B, GABRA3-COL6A6, GABRA3-VIT and GABRA3-FBN3.

In contrast, significant reduction of pro-tumorigenic RSPO1 and FGF16 upon treatment with BZDRs was elicited by GABRA3-shRNA and GABRA3-CRISPR/Cas9. Therefore, it would be interesting, prospectively, to delineate the physiological functions of RSPO1 and FGF16 in BZDR-associated signaling. The potential comprehensive interaction between GABA receptors, that could have significant implications on breast carcinogenesis, may be therapeutically manipulated to halt cancer advancement in BZDR-user patients, depending on which ECM candidates and /or GABA receptors or pro- /anti- tumorigenic factors are targeted in the networks.

### In silico-predicted molecular signaling of GABRA3 and ECMs indicates BZDR-driven BRCA progression.

**Fig. 6:**
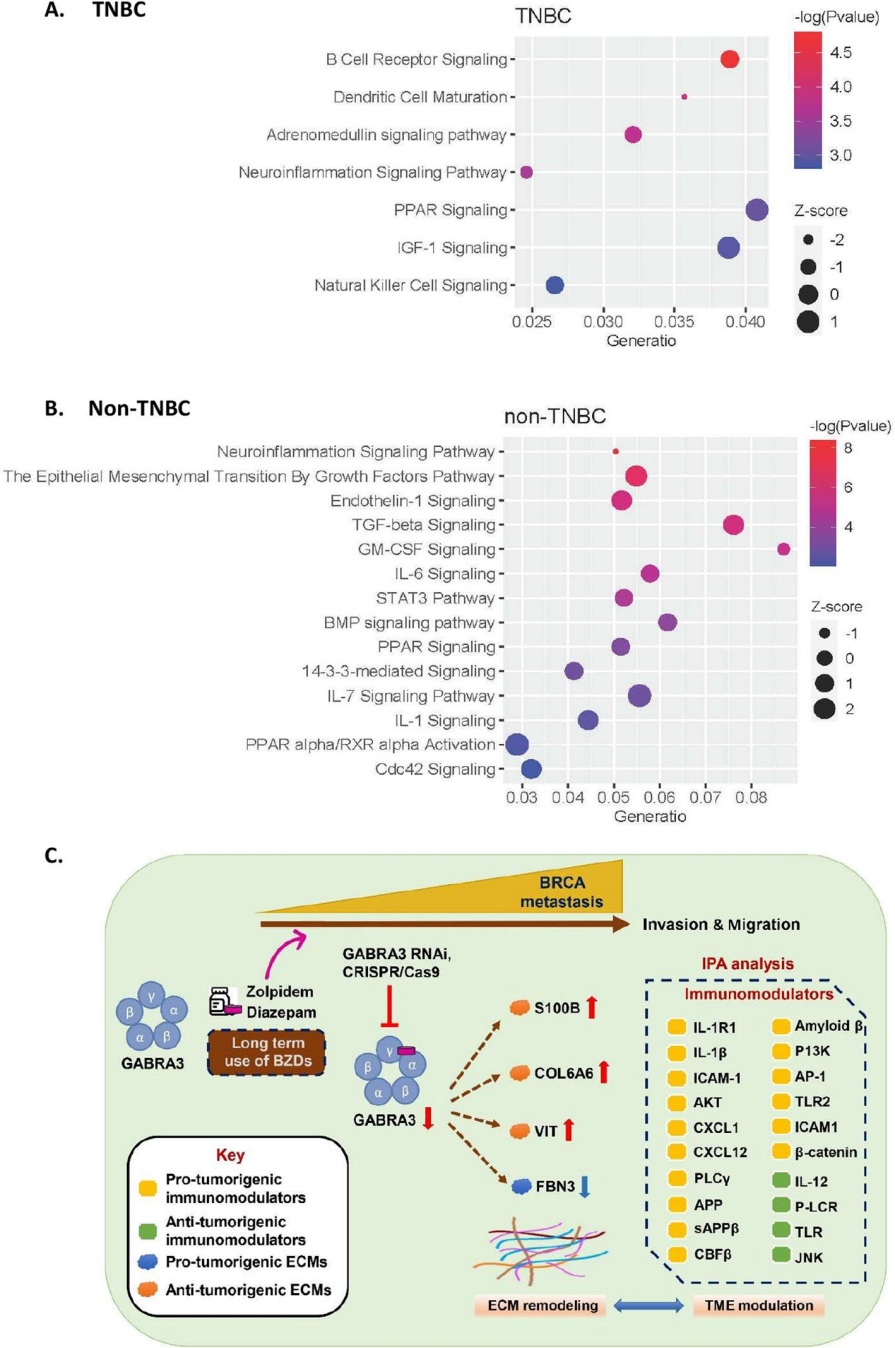
In silico-predicted molecular signaling of GABRA3 and ECMs indicates BZDR-driven BRCA progression. Canonical pathway analysis highlighted activated and suppressed pathways in BRCA, with *p* < 0.05 by IPA, based on the differential gene expression between early stage I, compared to later stages II, III, IV of BRCA for: **A** TNBC and **B** non-TNBC. **C** A schematic model shows that long-term use of BZDRs (Zolpidem and Diazepam) by BRCA patients promotes breast cancer advancement (indicated by cell invasion and migration) via modulating axes of GABRA3-ECMs. Treatment with BZDRs upregulate pro-tumorigenic GABRA3 and downregulate anti-tumorigenic ECMs (S100B, COL6A6 and VIT), as well as activate pro-tumorigenic ECM molecule, FBN3. GABRA3-shRNA and GABRA3-CRISPR/Cas9 dramatically restored effects observed above. Furthermore, IPA analysis indicates that multiple immunomodulators in the TME are involved in the GABRA3-ECM networks. Our results highlight that BZDR stimulates neuroinflammation signaling which plays key roles at the crossroad between specific ECMs and GABRA3

To reveal the potential signaling mechanisms associated with GABRA3 and ECMs in BRCA advancement, we performed Ingenuity Pathway Analyses (IPA) (Fig. 6A, B). The list of genes used in IPA is shown in the Supplementary Information (Supplementary Table S2). IPA predicted the modulation of several pathways associated with BRCA metastasis. There was significant activation of metastasis-promoting signaling pathways, including (i) for TNBC: PPAR signaling (z = 1) and IGF-1 signaling (z = 1) (Fig. 6A) and (ii) for non-TNBC: regulation of EMT by growth factor pathways (z = 1.41), endothelin-1 signaling (z = 1), TGF-β signaling (z = 1.13), IL-6 signaling (z = 0.38), STAT3 pathway (z = 0.38), BMP signaling pathway (z = 0.45), PPAR signaling (z = 0.45), 14-3-3-mediated signaling (z = 0.45), IL-7 signaling pathway (z = 2), IL-1 signaling (z = 1), PPARα/RXRα activation (z = 2) and Cdc42 signaling (z = 1) (Fig. 6B).

IPA consistently revealed strong inhibition of metastasis-suppression signalings, including adrenomedullin signaling pathway (z = − 2.24) in TNBC and GM-CSF signaling (z = − 0.82) in non-TNBC. Remarkably, the immune response pathways were suppressed in TNBC, including B cell receptor signaling, dendritic cell maturation and natural killer cell signaling (Fig. 6A). Furthermore, the IPA highlighted several pathways that GABRA3 and ECMs were significantly involved in BRCA metastasis, including the regulation of EMT by growth factor pathways, such as PI3K/AKT signaling and 14-3-3-mediated signaling in TNBC and BRCA regulation by Stathmin1 and estrogen-dependent BRCA signaling in non-TNBC (Supplementary Figure S2 in the Supplementary Information). For our analysis, we grouped these functions based on their mutual functional characteristics: (1) BRCA metastasis; (2) tumorigenic microenvironment, and (3) immunomodulation. Consistently, a recent study demonstrated that treatment with benzodiazepines was associated with poor clinical responses to chemoimmunotherapy in cancer patients as compared to individuals not receiving any psychotropic drugs [50]. Benzodiazepines was proposed to play immunosuppressive roles in cancer patients. Further interpretations of these data are deliberated in the discussion section, including the regulation of potential immunomodulators by BZDRs, in association with cancer progression.

## Discussion

Benzodiazepine is patented as an anti-cancer drug to target MDM2-p53 interaction [11]. Small molecule inhibitors such as BZDRs are proposed to block the p53-MDM2 interaction node, thus providing a promising anti-cancer candidate [51]. Despite being known to play a potential role in anti-cancer signaling, BZDRs are recently reported to associate with cancer risk [14, 52, 53] and our current findings from BRCA patient samples clearly demonstrated so. Of concern is that BRCA survivors suffering anxiety, are still prescribed BZDRs, with 30% patients using BZDRs over long-term (Fig. 1A) [54]. Here, we show for the first time, that treatment of mice with BZDRs exacerbates BRCA metastasis to the lungs, with localization in the tibia where bone microenvironment interaction and TME maintenance might occur (Fig. 1I) [25–27]. To confirm these in vivo results, we demonstrated, in vitro*,* that BZDRs promoted MDA-MB231 breast cancer cell progression by cell migration (Fig. 2D, E), as well as cell invasion (Fig. 5A, B). Altogether, our results showed that BZDRs promote BRCA advancement in vivo and in vitro. These results are also consistent with our clinical observation that long-term usage of BZDR is associated with poor survival rate of BRCA patients (Fig. 1B). Furthermore, using in vitro BRCA cell line studies, we elucidated the cellular and molecular mechanisms underlying how BZDRs exert pro-tumorigenic actions.

As an agonist of GABA receptors [55, 56], BZDRs are clinically relevant anti-convulsant drugs to treat seizure. However, BZDRs activate multiple networks, leading to diverse effects. We found that GABRP and GABRA3 are significantly induced by BZDR, resulting in BRCA progression. Ironically, GABRA3, which is recognized as a key pro-tumorigenic GABA receptor in BRCA [9, 57], is responsive to BZDR stimulation (Fig. 6C). This is consistent with our clinical observations that highly expressed GABRA3 is associated with lower five-year survival rate of BRCA patients (*p* = 0.012) (Supplementary Figure S3).

GABA/GABA receptors collaborate with ECM [22], presenting a highly dynamic platform of molecular candidates for the TME and cancer formation [58]. The GABA receptor-ECM network is envisaged to play critical roles in metastasis and effectively modulates stromal carcinogenesis [59]. We showed that 6 ECMs (RSPO1, FGF16, S100B, COL6A6, VIT/vitrin and FBN3) were intensively up/downregulated upon BZDR treatment, indicating a comprehensive dynamic balance of BZDR-mediated networks. This is supported by our findings that anti-tumorigenic S100B, COL6A6 and VIT were significantly suppressed by BZDRs (Fig. 5), presumably inducing GABRA3-mediated breast carcinogenesis. GABRA3 apparently pairs with various pro- or anti- tumorigenic ECMs, e.g. GABRA3-S100B, GABRA3-COL6A6, GABRA3-VIT and/or GABRA3-FBN3, to regulate cancer advancement (Fig. 6C). Expression of some of the ECM molecules in GABRA3 knock-down/-out studies persisted with BZDRs stimulation, which maybe due to incomplete knockdown, although GABRA3 knockdown using shRNA effectively reduced its gene expression by 85% in MDA-MB231 cells (Fig. 5C). Additionally, GABRA3 may not be the only receptor, and crosstalk with the other GABA receptors, such as GABRP may be occuring (Fig. 5D); alternatively, there could be other regulatory genes that may also contribute to ECM-associated BRCA tumor progression. Consistently, BZDR has been reported to increase other cancers, for example: 98% for brain, 25% for colorectal, and 10% for lung in BZDR users, compared to cancer patients who are non-BZDR users [19]. Supporting evidence proposed by a recent study showed that BZDR is associated with worse patient survival across multiple cancer types [60]. We suggest that BZDRs modulate different signaling pathways involved in inflammatory response and ECM signatures (Fig. 6) [60]. Altogether, our results indicate that there are common BZDR-mediated molecular pathway(s) that contribute to carcinogenesis.

By IPA, canonical pathway analysis shown in Fig. 6A, B and Supplementary Fig. S2, revealed the activation of metastasis-promoting signaling pathways as well as inhibition of metastasis-suppression signalings at networks of GABRA3 and ECMs. Consistently, we observed signaling pathways which collaborate with GABRA3 and ECMs (Supplementary Fig. S4 in the Supplementary Information). IPA did not predict partnership between other GABA receptors and ECMs networks, again affirming that GABRA3 is the key player in BRCA advancement. The in silico prediction highlighted the neuroinflammation pathway, which may be the key signaling node at the crossroad between our identified ECMs and GABA receptors, mediated by BZDR neurotherapy.

Several immunomodulatory molecules, including IL-1R1, IL-1β, ICAM-1, AKT, CXCL1, CXCL12, PLCγ, APP, sAPPβ, CBFβ, Amyloid β, P13K, AP-1, TLR2, β-catenin, ICAM1, IL-12, P-LCR, JNK, TLR were identified (Fig. 6C). Such immunomodulators are known to be associated with TME maintenance [61]. For example, IL-1β promotes BRCA metastasis via Wnt signaling [62]. Suppression of IL-1β-NFκB/CREB-Wnt pathway is known to prevent both BRCA metastasis to bone in vivo, and colony formation of cancer stem cells in the bone microenvironment in vitro. Additionally, a current study of triple negative BRCA (TNBC) indicated that a sub-population of fibroblasts, namely ECM cancer-associated fibroblasts were enriched. These fibroblasts attract macrophages that promote fibrosis and TGF-β and IL-1β secretion. IL-1β is a pro-inflammatory molecule and TGF-β signaling play important roles in ECM remodeling and immunomodulation [63]. Pro-tumorigenic ICAM-1 enhances EGFR activation and JAK1/STAT3 signaling to drive epithelial-to-mesenchymal transition and metastasis in BRCA [64]. CXCL12 promotes BRCA cell migration and invasion towards lymphatic vessels via CCR7 signaling [65]. CXCL12 facilitates CCR7 homodimer formation, supporting lymph node metastasis by TME modulation. On these premises, our findings provide a platform for prospective intensive elucidation of whether and how these immunomodulatory molecules may participate in networks of ECMs/GABA receptors in BZDR-associated TME. However, further studies are required in future, to validate signaling pathways identified by IPA, particularly those which are driven by GABRA3 and ECM remodeling. Such studies should provide further insights on how immunomodulation may be co-opted to improve therapeutic programs involving BZDR-treatment of patients with cancer. For example, the GABRA3-ECM pathway may be therapeutically manipulated to halt cancer advancement in BZDR-user patients.

## Novelty and impact

Long-term clinical use of BZDRs for treatment of insomnia and anxiety significantly increased the mortality rate of BRCA patients. We found BZDRs to promote cancer progression with increased mortality rate in BRCA patients. Here, we provide in vitro and in vivo evidence coherent with our clinical findings, to support BZDR-driven BRCA metastasis. We revealed that BZDRs target the GABRA3-ECM signaling pathways to promote cancer advancement through immune modulators and changes in the tumor microenvironment. Our findings have implications on improving preventive care and therapeutic programs involving BZDR-treatment of BRCA patients.

## Supplementary Information

Supplementary material 1.

## Data Availability

Data sharing is not applicable to this article as no datasets were generated or analysed during the current study.

## Abbreviations

ADAMTS8: ADAM metallopeptidase with thrombospondin type 1 motif 8
AKT: Protein kinase B
ATCC: American type culture collection
BRCA: Breast cancer
BZDRs: Benzodiazepines and related Z-drugs
COL6A6: Collagen type vi alpha 6 chain
CX3CL1: Chemokine (C-X3-C motif) ligand 1
CXCL12: C-X-C motif chemokine ligand 12
DESeq2: Differential gene expression analysis based on the negative binomial distribution
DMEM: Dulbecco's modified eagle medium
DMSO: Dimethyl sulfoxide
ECM: Extracellular matrix
EMT: Epithelial mesenchymal transition
FBN3: Fibrillin 3
FBS: Fetal bovine serum
FGF16: Fibroblast growth factor 16
GABAA: Amino butyric acid A
GABRA1/2/3/4/5/6: Gamma-aminobutyric acid receptor subunit alpha-1/2/3/4/5/6
GABRB1/2/3: Gamma-aminobutyric acid type A receptor subunit beta 1/2/3
GABRD: Gamma-aminobutyric acid receptor subunit delta
GABRE: Gamma-aminobutyric acid type A receptor subunit epsilon
GABRG1/2/3: Gamma-aminobutyric acid Type A receptor subunit gamma 1/2/3, GABRGQ: gamma-aminobutyric acid type A receptor subunit theta
GABRP: Gamma-aminobutyric acid receptor subunit pi
HER2: Human epidermal growth factor receptor 2
ICAM-1: Intercellular adhesion molecule-1
IL-12: Interleukin 12
IL-1R1: Interleukin 1 receptor, type I
IL-1β: Interleukin 1 beta
IPA: Ingenuity pathway analysis
MDM2: Mouse double minute 2 homolog
p53: Tumor protein p53
P-LCR: Platelet -larger cell ratio
qRT-PCR: Real-time quantitative reverse transcription PCR
RNAi: RNA interference
RSPO1: Human r-spondin1
S100B: S100 calcium binding protein B
SHH: Shuang-Ho Hospital
shRNA: Short hairpin RNA
TCGA: The cancer genome atlas
TCR: Taiwan Cancer Registry
TDR: Taiwan Death Registry
TME: Tumor microenvironment
TMUCRD: Taipei Medical University Clinical Research Database
TMUH: Taipei Medical University Hospital
TNBC: Triple negative breast cancer
VIT: Vitrin
WFH: Wan-Fang Hospital
