# Supplementary material for: Long-term treatment with benzodiazepines and related Z-drugs exacerbates breast cancer: clinical evidence and molecular mechanisms
Source: Cell Mol Biol Lett. 2025 Jun 29;30:75. doi: 10.1186/s11658-025-00752-4 (PMC12206364; doi:10.1186/s11658-025-00752-4)
Supplement: Supplementary file 1 — Supplementary material 1. [file 11658_2025_752_MOESM1_ESM.pdf]

Supplemental Data

Supplementary Table S1.

A. Average expression value

	GABRA3	GABRD	GABRP	GABRA2	GABRE	GABRG1
Normal	0.868	18.544	16026.494	55.601	1920.895	12.215
stage I	11.830	189.756	10413.123	12.899	295.429	1.697
stage II	35.181	206.439	9723.457	20.806	370.145	2.013
stage III	35.277	214.628	5239.819	10.743	299.941	2.703
stage IV	117.567	234.122	10981.531	10.330	527.898	0.710

B. Standard deviation

	GABRA3	GABRD	GABRP	GABRA2	GABRE	GABRG1
Normal	2.556	25.026	11972.475	56.806	1993.472	15.594
stage I	45.530	239.893	30249.157	47.019	572.000	2.509
stage II	123.468	227.373	23894.242	94.018	1047.556	5.055
stage III	132.977	176.920	17395.268	36.214	695.032	7.479
stage IV	286.638	216.947	29677.933	24.399	1325.859	1.702

Supplementary Table S1. The expression levels of identified GABA receptors in primary BRCA tissues. To better understand the physiological relevance of GABA receptors in BRCA development and progression, we retrospectively examined expression profiles of GABA receptors from TCGA database. (**A**) shows average value and (**B**) shows standard deviation. Among 16 GABA receptors examined, 2 GABA receptors, GABRA3 and GABRD, were significantly upregulated in breast carcinoma tissues compared to normal breast tissues (* p<0.05; ** p<0.01). On the other hand, 4 GABA receptors (GABRA2, GABRE, GABRG1 and GABRP) were significantly downregulated in BRCA tissues (* p<0.05; ** p<0.01). Details of the expression levels of these GABARs are shown here.

Supplementary Table S2

A. Triple Negative Breast Cancer (TNBC)

Triple Negative Breast Cancer (TNBC)						
FGB	MUSK	FGF3	CBLN2	GC	IL1F6	COL11A2
IL9	SERPINA7	FGL1	ANGPTL7	VWC2	FNDC7	RSPO1
C1QL1	HAPLN1	SERPINA4	COL28A1	GPC5	ZPLD1	PRSS12
FGF16	CADM3	ADAMTS8	COL6A6	VIT	CST1	EXTL1
TNFSF13B	GPD1	CCL22	ISM1	S100B	ERBB4	PAPPA2
AKT3	CD58	ANGPT1	BTC	CXCL11	SPARCL1	FBN3
A2M	COL11A1	IL12B	ITPR2	XYLT1	COL4A3	COLEC12
PLCG2	CX3CL1	EDN1	FGF1	MAP2	COL27A1	SERPING1
CTSO	CRIM1	VWA5A	GABRP	IL6ST	TIMP3	PDGFD
IL1R1	HAPLN3	TNFSF4	MXRA5	PLXNB1	RUNX1	GAB1
FRS2	CAMK2D	MDM2	SP1	NUDT16L1	ELK1	CLPP
MAP2K2	LONP1	RPS6KB2	COLGALT1	B3GAT3	TRIB3	APOC2
RTN4RL1	PRG3					

B. Non-Triple Negative Breast Cancer (non-TNBC)

Non-Triple Negative Breast Cancer (non-TNBC)						
GABRA3	CCL24	IL1B	CHSY1	IL13	HMMR	FOS
CLC	RTN4RL1	FGF16	CTSA	INSL5	NTN4	NANOG
FCN1	CLEC10A	CSF2	CLEC4C	IL5	ITPR1	CALM1
RRAS2	SERPINB9	NPNT	SERPINB8	ICAM1	ADAM9	RUNX1
BMPER	CSF1	ADAMTS8	MUC16	MYDGF	SERPINB2	MAPK13
COL6A6	MMP19	PLCB4	PAPLN	SMAD7	ITGAX	GDF1
ARAF	ADAMTS9	ASPN	APP	CXCL3	SRGN	PPARG
PIK3CB	WNT16	HAS1	ADAMTS6	PRG2	P4HA2	LRG1
THBD	TNC	SULF2	PTPRS	RSPO1	CCL3L3	LGALS8
CHRDL2	TGFBR3	COL9A1	DMBT1	VWA5A	CCL8	MAP2
PLXNA3	PRG3	SERPINB3	SEMA4F	ITGA9	WNT3A	MAPK12
S100A7	CSTA	SERPINB4	SMO	VIT	ELSPBP1	C17orf58
PLXNB3	RNASE3	TPSG1	PDGFB	EGLN1	BTRC	CRISPLD2
ARHGEF12	MAPK14	SEMA4C	CAMK2G	CALCRL	TLR2	S100B
TSPEAR	CAMK2B	INHBA	S100Z	FGF20	NDST1	CD44
CCL17	SERPINA5	ACAN	IL20	MEGF11	RELN	CXCL12
CSF3	FBN3	VWA2	CTNNB1			

Supplementary Table S2. The list of genes applied in IPA analysis. To better understand the molecular pathways in the networks between GABRA3 and ECMs in BRCA progression, genes selected here are applied to IPA analysis. Both (**A**) triple-negative BRCA and (**B**) non- triple-negative BRCA are analyzed. Genes listed here are considered as significant (p<0.05) between early stage I, compared to later stages II, III, IV of BRCA.

Supplementary Figure S1

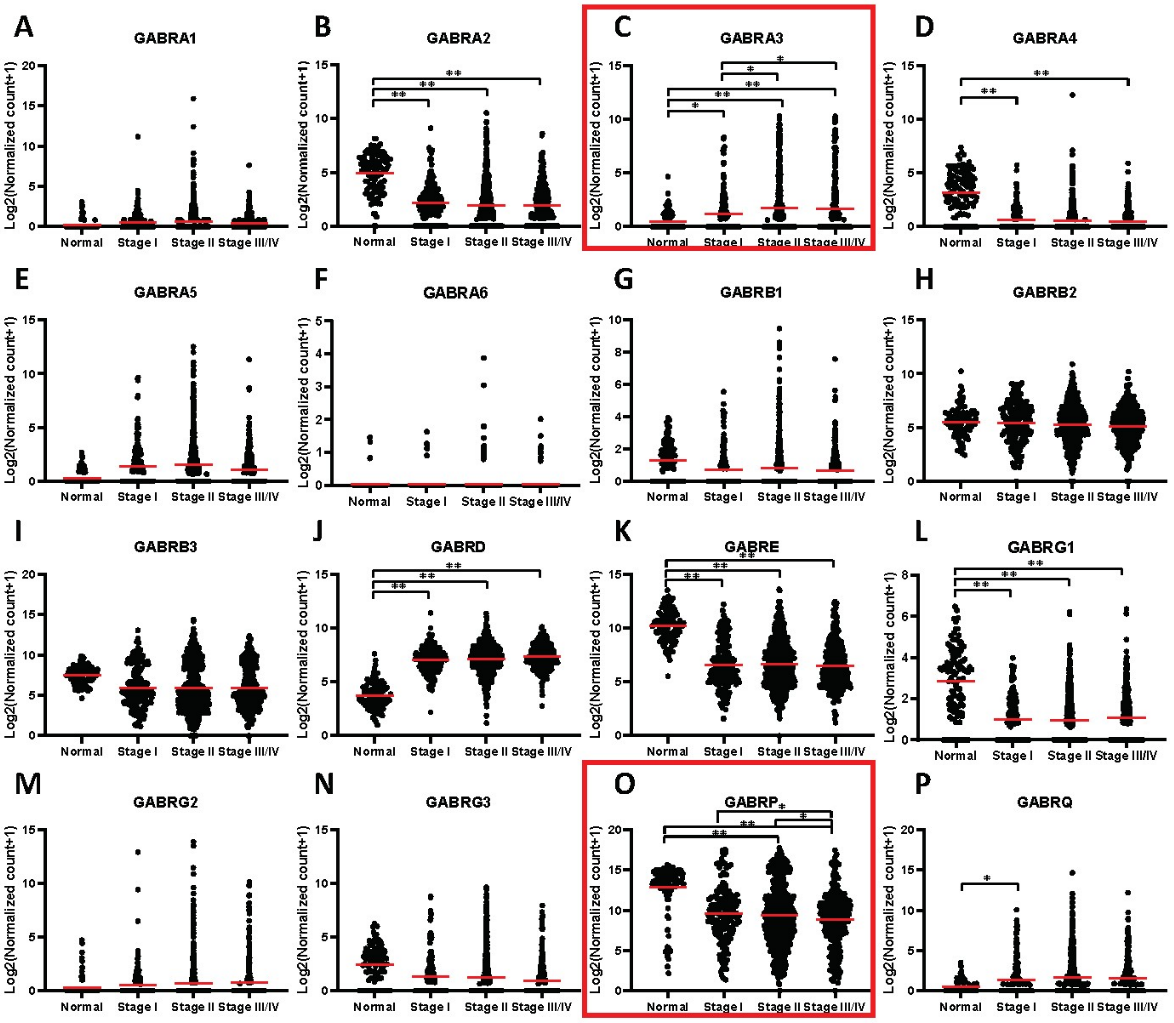

Supplementary Figure S1. BZDRs significantly upregulated GABRA3 expression which is associated with BRCA staging. To characterize the expression profiles of GABA receptors in human breast cancer, we analyzed The Cancer Genome Atlas (TCGA) database (see details in Materials and Methods). Expression profiles with log scale are illustrated here. In total, 1189 primary samples were examined, including 112 normal breast tissues and 1077 carcinoma breast tissues (Stage I: 182, Stage II: 624, Stage III: 251 and Stage IV: 20). 16 GABA receptors were identified, including (A) GABRA1, (B) GABRA2, (C) GABRA3, (D) GABRA4, (E) GABRA5, (F) GABRA6, (G) GABRB1, (H) GABRB2, (I) GABRB3, (J) GABRD, (K) GABRE, (L) GABRG1, (M) GABRG2, (N) GABRG3, (O) GABRP and (P) GABRQ. The upregulated and downregulated expression of GABRA3 and GABRP, indicated by red boxes (panels C & O), respectively) appeared to be associated with staging events. In advanced status of the disease (stage III/IV), GABRA3 was significantly over-expressed compared to early stage I. However, GABRP was further reduced in stage III/IV of BRCA, compared to early stage I.

Supplementary Figure S2

A. Triple Negative Breast Cancer (TNBC)

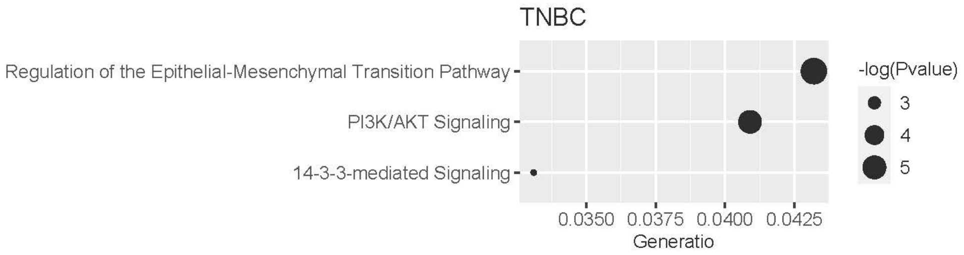

B. Non-Triple Negative Breast Cancer (non-TNBC)

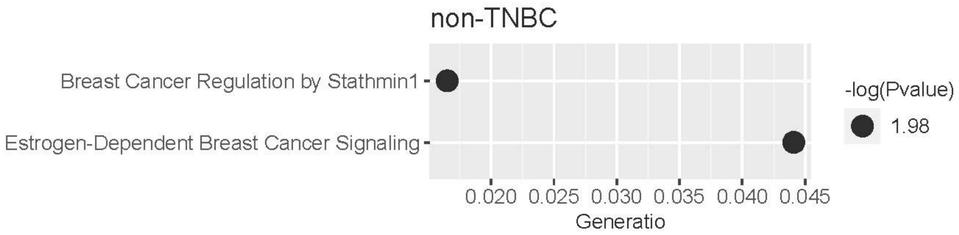

Supplementary Figure S2. Canonical Pathway analysis by IPA. Prediction of participating pathways in BRCA, with $p<0.05$, based on the differential gene expression between early stage I, compared to later stages II, III, IV of BRCA. (**A**) TNBC and (**B**) non-TNBC.

Supplementary Figure S3

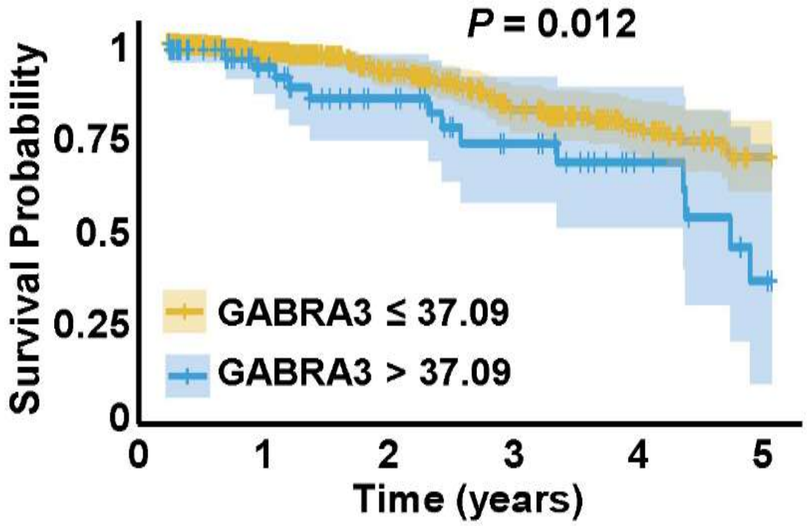

Supplementary Figure S3. The five-year overall survival for BRCA patients with high- vs low- GABRA3 expression levels. In total, 893 BRCA patients (stage II: 623, stage III: 250, and stage IV: 20) were examined, and divided into yellow group: normalized count ≤ 37.09, and blue group: normalized count > 37.09. Yellow group (n =786) consists of patients with low-expression of GABRA3, whereas blue group (n=107) consists of patients with highly-expressed GABRA3. The X-axis represents time in years, while the Y-axis indicates the overall survival probability. Kaplan-Meier estimates show the survival probability for each group. Patients with higher expression levels of GABRA3 showed lower five-year survival rate than patients with lower expression levels (*p*=0.012).

Supplementary Figure S4

A. Triple Negative Breast Cancer (TNBC)

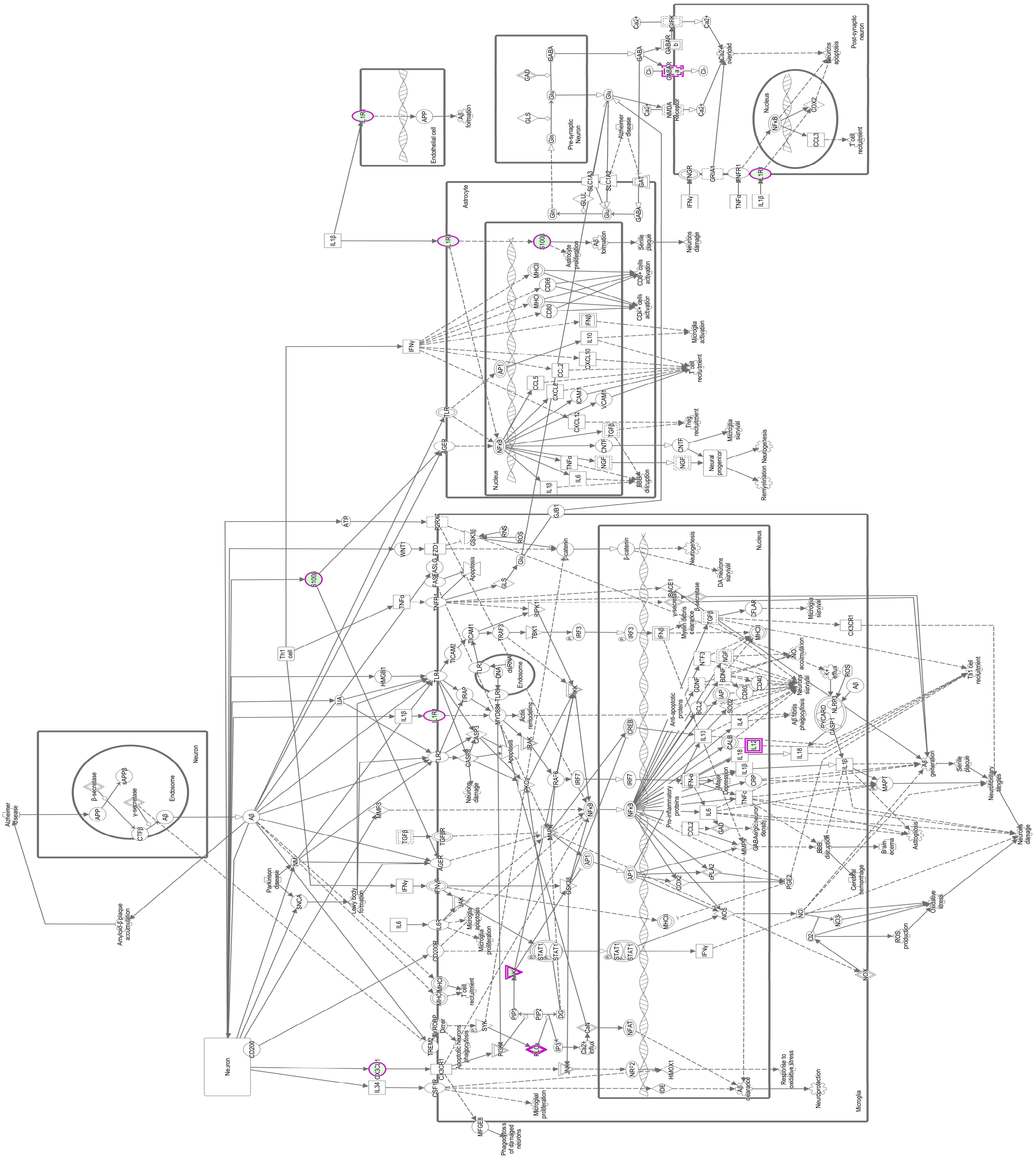

B. Non-Triple Negative Breast Cancer (non-TNBC)

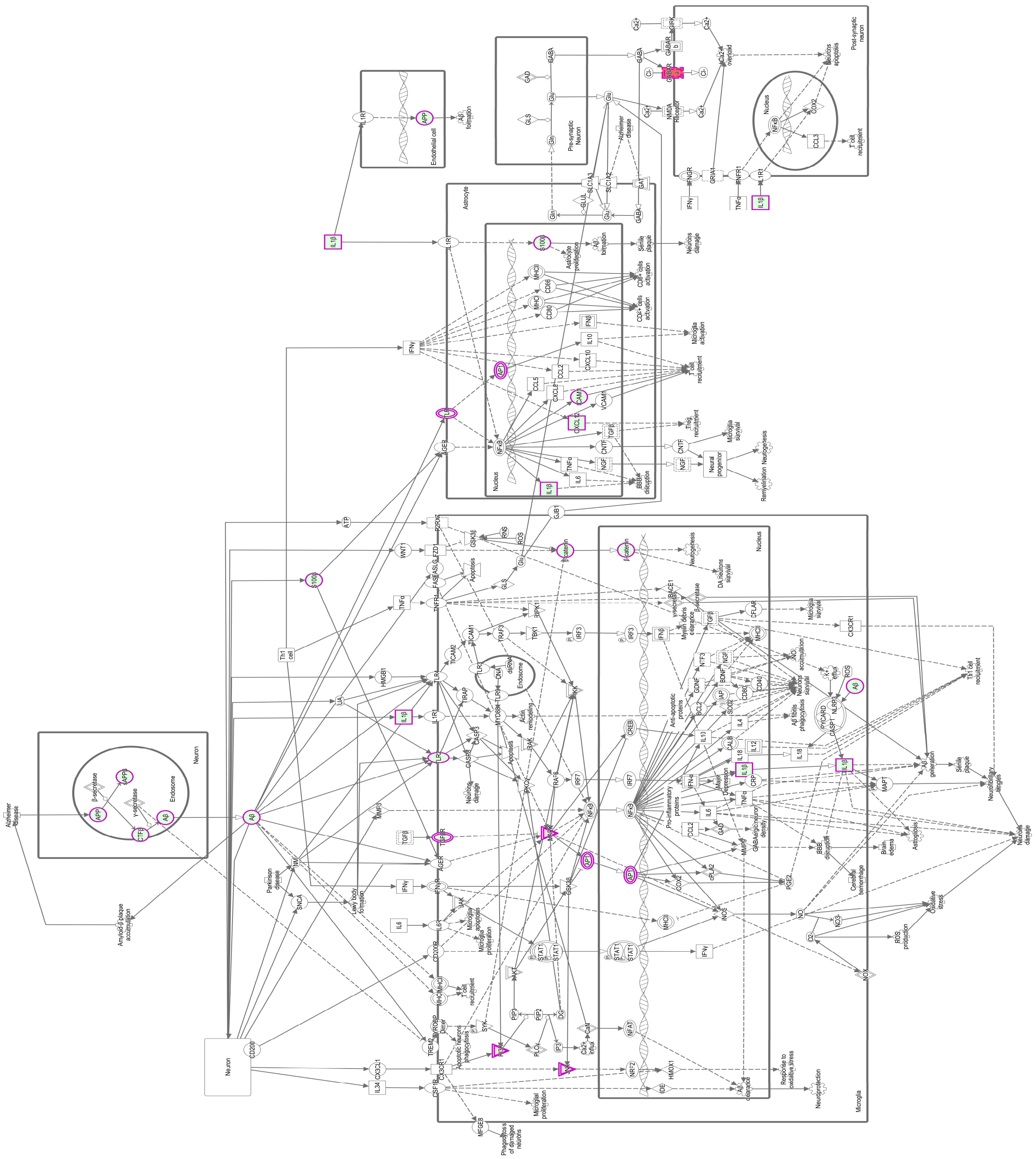

Supplementary Figure S4. IPA analysis of GABRA3-mediated signaling and key ECM molecules involved in BRCA progression. To better understand the potential regulators involved in GABRA3 and ECM associated signalings, gene network analysis was performed using Ingenuity® Pathway Analysis (IPA®, QIAGEN Redwood City) software. Genes that are significantly differentially expressed serve as input for canonical pathway analysis. Both **(A)** triple-negative BRCA and **(B)** non- triple-negative BRCA were analyzed. Several immune-modulators emerged, including pro-tumorigenic factors: IL-1R1, IL-1β, ICAM-1, AKT, CXCL1, CXCL12, PLCγ, APP, sAPPβ, CBFβ, Amyloid β, P13K, AP-1, TLR2, β-caterin, ICAM1; as well as anti-tumorigenic factors: S100B, IL-12, P-LCR, JNK, TLR.

Supplemental Experimental Procedures

Source of patient data from TMUCRD

This study obtained data from the TMUCRD from January 1, 2008 to December 31, 2020 (n=6464). The database combines the comprehensive clinical data from three medical centers: Taipei Medical University Hospital (TMUH), Wan-Fang Hospital (WFH), and Shuang-Ho Hospital (SHH)) in the North of Taiwan. Taiwan's Health Promotion Administration and Ministry of Health and Welfare link and manage the database to the Taiwan Cancer Registry (TCR) and Taiwan Death Registry (TDR) databases, established in 1979. Furthermore, the TMUCRD contains the electronic medical record data of more than four million patients from 1998 to 2021, including structured and unstructured data. This clinical study has been approved by the Joint Institute Review Board of Taipei Medical University, Taipei, Taiwan (IRB: N202201089). The data were anonymized before further analysis.

Clinical population examined from TMUCRD

We conducted a retrospective study to identify all female patients diagnosed with primary breast cancer (International Classification of Disease for Oncology, third edition [ICD-O-3] codes C50) from January 1, 2009, to December 31, 2019, in the TMUCRD database (n=3914). We excluded subjects who were younger than 20 years old and those who did not have any medical history in the three hospitals. We also excluded the patients who were followed-up for less than 3 months (i.e., 90 days) after breast cancer diagnosis. In total, 3510 patients are included in this study cohort, including n=2457 non-BZDR group and n=1053 BZDR group.

BZDR classification and exposure

TMUCRD has recorded information on all prescribed drugs dispensed from three affiliated hospitals. BZDRs were classified as ATC code N03AE (i.e., Antiepileptics);

codes for anxiolytics medications were N05BA (mostly diazepam), and hypnotics and sedatives were N05CF (mostly zolpidem and zopiclone). We defined BRCA diagnosis date as the index date, and the outcome of the study was 5-year survival after the index date. Medical records were reviewed for in-hospital deaths, and the TDR was referred to in order to confirm the death status from within and outside hospitals. The data were censored on the outcome date, as a lack of follow-up (e.g. terminated national health insurance), or at the end of the study on December 31, 2020. The use of BZDR was recorded both before and after the date of a cancer diagnosis. In our analysis, we considered whether patients had ever used BZDRs for a duration exceeding one month subsequent to their cancer diagnosis, i.e., 30 days within a year, as compared to those who did not use BZDRs. Furthermore, patients who were prescribed BZDRs less than three times (90 days per year) were also excluded in the study. In addition, patients who had been prescribed BZDRs for a period extending beyond 30 days in a year before their cancer diagnosis, were classified as regular BZDR users. This information was then used in further analysis for outcome adjustment.

Cell lines and reagents

Human breast cancer cell lines (MCF7, MDA-MB231) and a control non-carcinoma breast cell line (MCF10A) were obtained from American Type Culture Collection (ATCC). All human cell lines used in this work are mycoplasma-free cells and are authenticated using STR profiling within the last three years. Cell lines were maintained as previously described (1). The MCF7 (RRID:CVCL_0031) and MDA-MB231 (RRID:CVCL_0062) were cultured in complete DMEM medium (Gibco). MCF10A (RRID:CVCL_0598) was cultured in complete DMEM/F12 medium (Gibco). Both of the complete media were supplemented with 10 % FBS (Thermo Scientific), 100 U/ml penicillin and 100 μg/ml streptomycin (Invitrogen).

Quantitative real-time PCR (qRT-PCR)

qRT-PCR was performed as previously described (2). The primers were human RSPO1 (146 bp product): sense, 5'-ACACTTCCCAGCATCTGAGACCAA-3'; antisense, 5'- TGCTGAACAGGATGGGAAGAAGGT-3'; human GABRA3 (165 bp product): sense, 5'-CCGTCTGTTATGCCTTTGTATT-3', antisense, 5'-TGTTGAAGGTAGTGCTGGTTTT-3'; human GABRP (120 bp product): sense, 5'-CGACCGTGTTATCAATGACC-3'; antisense, 5'- CCCCAAACACAAAGCTAAAGCA-3'; human FGF16 (68 bp product): sense, 5'-CACGGCTTCTCCTCGTCTCT-3', antisense, 5'-AGGCGCTCGTTCAGGAAA-3'; human ADAMTS8 (138 bp product): sense, 5'-CCGCCACCCAGAGCACTA-3'; antisense, 5'-TCGATCACGGAGCAGCTTTT-3'; human COL6A6 (195 bp product): sense, 5'-CCCAGGCCACAGATTTCCAT-3', antisense, 5'-TCCCACCCATCTGCCTGATA-3'; human VIT (105 bp product): sense, 5'- GGTCTGCGACACTGACCGCC-3', antisense, 5'-CGGAAGTTGCCCGTCCCCAC-3'; human S100B (189 bp product): sense, 5'-AGGGAGACAAGCACAAGCTG-3', antisense, 5'-CGTGGCAGGCAGTAGTAACC-3'; human FBN3 (174 bp product): sense, 5'-ACCTGGACGAATGCACCTC-3', antisense, 5'-CTGAGCTGACCAGGGTGAAG-3'; human GAPDH (131 bp product): sense, 5'-GTCTCCTCTGACTTCAACAGCG-3', antisense, 5'-ACCACCCTGTTGCTGTAGCCAA-3'. All expression values were normalized based on GAPDH as an endogenous control.

shRNA RNAi knockdown of GABRA3 in BRCA cells

GABRA3 shRNA clones (ID numbers: TRCN0000061208, TRCN0000061209, TRCN0000061210, TRCN0000061211, TRCN0000061212, TRCN0000420690 and TRCN0000426309) and control (scrambled) shRNA were purchased from (Academia Sinica, Taipei, Taiwan). The transfection experiments were performed as previously described (3). Briefly, shRNA was transfected into MCF10A or MDA-MB231 cells (at a

cell density of $5x10^5$ cells per well of a 6-well plate) using 7.5 µl TransIT-X2 Transfection Reagent (Mirus) with 25 nM of shRNA per well. Antibiotics, puromycin, was added at a concentration of 2 µg/ml to the wells 48 h after transfection to select for positively transfected cells. Then, the cells were selected for 2 months before experiments were carried out and were continuously grown in selection media. The efficiency of RNAi knockdown was determined by real-time PCR of GABRA3 mRNA (shown in Figure 4C).

The CRISPR/Cas9 system

We used a dual gRNA approach to knockout the full-length sequence of GABRA3 by CRISPR/Cas9 system (Ubigene, Guangzhou, China). The study was performed as previously described (4). Briefly, dual gRNA-vectors were transfected into MDA-MB231 cells. One week later, the transfected cells were subjected to puromycin (1 µg/ml) selection; surviving cells were sorted into 96-well plates and expanded into 12-well plates. Potential clones were further verified by qRTPCR.

Cell growth assay

The cell growth was measured by Trypan Blue dye exclusion test. The study was performed as previously described (1). Specifically, to assess total cell number, cells were trypsinized and resuspended in equal volumes of culture medium and trypan blue dye (0.4% solution; Gibco, Waltham, MA, USA). The cells were counted using an improved Neubauer hemocytometer.

Cell migration assay

Cell migration assay was carried out 7 days after treatment of MCF7 and MDA-MB231 cells with BZDRs or DMSO control. The study was performed as previously described (2). Briefly, after the abovementioned treatment the cells were rinsed with PBS, and

harvested using 0.05% Trypsin-EDTA (Invitrogen). The cells were then plated into a 2-well Culture-Insert (ibidi, Martinsried, Germany) according to the manufacturer's instructions. The Culture-Inserts were removed after 16 h, and cell migration was monitored over the indicated time-scale.

Cell invasion assay

Cell invasion study was performed as previously described (1). Briefly, invasion chambers with 8-μm pores in 24-well plates (Corning, Discovery Labware, Inc., Bedford, MA, USA) were used. 192 h after BZDR treatment, the cells were plated onto the chambers. Subsequently, the cells were detached with 0.05 % Trypsin-EDTA, resuspended in conditioned medium (10 % FBS) and added to the upper compartment of the chambers, according to the manufacturer's instructions. After 20 h of incubation at 37 °C, the cells on the upper chamber were completely removed by wiping with a cotton swab, and then the filter was fixed with 100 % methanol and stained with 1% Toluidine Blue in 1% borax. Cells that had migrated from the upper to the lower side of the filter were imaged and counted with a light microscope (5 fields/filter).

IPA Analysis of TCGA dataset

The TCGA breast carcinoma cohort (TCGA-BRCA) transcript dataset was downloaded from the R package, TCGAbiolinks (5). All raw counts were normalized using DESeq2. In total, 1189 primary tissues were used in this study, including 1077 carcinoma tissues and 112 normal tissues. To determine whether specific genes showed statistically significant differential expression between normal and different tumor stages, a Student's t-test was performed. The p value < 0.05 was used as a threshold to define genes that showed statistically significant differential expression. The significant genes serve as input for loading into Ingenuity Pathway Analysis (IPA) to conduct pathway analysis. The value of -log (p-value) is greater than 1.3, as specific and significant pathways were selected to create a dot plot using the R package

ggplot2. The z-score from IPA forecasts either activation or inhibition of a pathway or gene; a negative z-score signifies overall inhibition, whereas a positive z-score indicates overall activation.

Statistical Analysis

Hazard ratios (HRs) with 95% confidence intervals (CI) associated with statin use were computed using Cox proportional hazards regression in the competing risk of death after adjusting for age, CCI scores, tumor stage, tumor size, and ER/PR/HER2 status. All TMUCRD data management was performed using SAS v.9.3 software (SAS Institute Inc).

Supplementary References

1. Chang SC, Hsu W, Su EC, Hung CS, Ding JL. Human FBXL8 Is a Novel E3 Ligase Which Promotes BRCA Metastasis by Stimulating Pro-Tumorigenic Cytokines and Inhibiting Tumor Suppressors. Cancers. 2020;12(8).
2. Chang SC, Hung CS, Zhang BX, Hsieh TH, Hsu W, Ding JL. A Novel Signature of CCNF-Associated E3 Ligases Collaborate and Counter Each Other in Breast Cancer. Cancers. 2021;13(12).
3. Moore CB, Guthrie EH, Huang MT, Taxman DJ. Short hairpin RNA (shRNA): design, delivery, and assessment of gene knockdown. Methods Mol Biol. 2010;629:141-58.
4. Liang J, Li G, Liao J, Huang Z, Wen J, Wang Y, et al. Non-coding small nucleolar RNA SNORD17 promotes the progression of hepatocellular carcinoma through a positive feedback loop upon p53 inactivation. Cell Death & Differentiation. 2022;29(5):988-1003.
5. Colaprico A, Silva TC, Olsen C, Garofano L, Cava C, Garolini D, et al. TCGAbiolinks: an R/Bioconductor package for integrative analysis of TCGA data. Nucleic Acids Res. 2016;44(8):e71.
